# Photothermal‐Responsive Soluble Microneedle Patches for Meibomian Gland Dysfunction Therapy

**DOI:** 10.1002/advs.202413962

**Published:** 2025-01-30

**Authors:** Fei Yu, Xuan Zhao, Qian Wang, Yifei Niu, Peng Xiao, Jinze Zhang, Keyi Fei, Yuancong Huang, Liu Liu, Po‐Han Fang, Xinyue Du, Weihua Li, Dalian He, Tingting Zhang, Saiqun Li, Jin Yuan

**Affiliations:** ^1^ Beijing Tongren Eye Center Beijing Tongren Hospital Capital Medical University Beijing Key Laboratory of Ophthalmology & Visual Sciences Beijing 100730 China; ^2^ State Key Laboratory of Ophthalmology Zhongshan Ophthalmic Center Sun Yat‐sen University Guangzhou 510623 China; ^3^ Sun Yat‐sen Memorial Hospital Sun Yat‐Sen University Guangzhou 510020 China; ^4^ National Clinical Research Center for Ocular Diseases Eye Hospital Wenzhou Medical University Wenzhou 325027 China

**Keywords:** meibomian gland dysfunction, PPAR‐γ, rosiglitazone, soluble microneedle patch, transdermal drug delivery

## Abstract

Meibomian gland dysfunction (MGD) is a leading cause of evaporative dry eye disease, presenting a challenge for targeted treatment. Traditional topical ocular drug delivery methods often fail to effectively reach the meibomian glands (MGs). To address this, the study has developed a soluble microneedles (MN) patch comprising poly(vinyl alcohol), cyclodextrin modified polyacrylic acid, and new indocyanine green. This innovative MN patch facilitates the transdermal release of peroxisome proliferator‐activated receptor gamma (PPAR‐γ) agonists, such as rosiglitazone in response to near‐infrared ray induced temperature changes. By safely optimizing temperature, the patch effectively liquefied meibum lips, thereby alleviating duct obstruction while releasing the drug. MN patches exhibit sufficient mechanical strength for effective skin penetration, and its biosafety for eyelid application has been rigorously assessed in vitro and in vivo. The therapeutic efficiency of rosiglitazone loaded MN (ROSI‐MN) treatment for MGD is evaluated in high‐fat mice. After three months of treatments, ROSI‐MN administration significantly alleviated MGD clinical manifestations, including ocular surface damage, lipid deposits, glandular hypertrophy, and inflammatory infiltration, ultimately improving the microstructure and biofunction of MGs. In conclusion, the soluble MN patches hold promise as an effective drug delivery strategy for treating ocular surface diseases beyond MGD.

## Graphical abstract

This study provides a soluble microneedles (MN) patch that facilitates the transdermal release of rosiglitazone in response to near‐infrared ray induced temperature changes. The MN shows therapeutic efficiency for meibomian gland dysfunction (MGD) in high‐fat mice. It offers promising drug delivery strategy for treating ocular surface diseases beyond MGD.

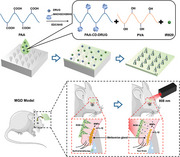

## 1 Introduction

Dry eye has emerged as a widespread and persistent ocular surface disease, causing not only discomfort but also visual dysfunction, significantly compromising overall quality of life.^[^ ^1^ ^]^ The primary culprit behind dry eye is tear film instability, primarily attributed to a reduction in the lipid layer, which forms the outermost part of the tear film.^[^ ^2^ ^]^ Tear lipids prevent tear film evaporation and create a smooth optical surface between the air and the aqueous layer, while also serving as a protective barrier against microbial agents and organic substances.^[^ ^3^ ^]^ These lipids are secreted by meibomian glands (MGs), which are a series of vertically aligned lipid‐producing glands in the upper and lower eyelids of mammals, located within the tarsal plate.^[^ ^4^ ^]^ Meibomian gland dysfunction (MGD) is the primary cause of evaporative dry eye, characterized by terminal duct obstruction and/or qualitative/quantitative changes in the glandular secretion.^[^ ^5^ ^]^ The development of MGD is influenced by various endogenous and exogenous factors, including aging, gender, lifestyle, and medications.^[^ ^5^, ^6^ ^]^

The primary goal of MGD treatment is to restore the flow of MG secretions and stabilize the tear film.^[^ ^6b^ ^]^ Numerous treatment options for MGD have emerged in recent years, heat therapy—such as warm compresses or devices like LipiFlow and Mibothermoflo—being highly regarded.^[^ ^6^, ^7^ ^]^ The melting point of meibomian lipids typically ranges from around 19 to 32 °C, ensuring them liquid at body temperature.^[^ ^8^ ^]^ However, in MGD, altered meibum composition can raise its melting point, necessitating temperatures above 40 °C to liquefy the obstructive materials.^[^ ^9^ ^]^ This principle underpins the basis of heating therapy in MGD management.

Beyond physical therapies, increasing attention is being given to medical treatments for MGD. Peroxisome proliferator‐activated receptors (PPARs) are lipid‐sensitive nuclear receptors involved in various physiological functions, including lipid metabolism and the differentiation of adipocytes and sebocytes.^[^ ^10^ ^]^ Previous studies have observed the expression of PPAR‐γ in MGs.^[^ ^11^ ^]^ Furthermore, downregulation of the PPAR‐γ pathway has been linked to MGD development.^[^ ^12^ ^]^ Therefore, targeting PPAR‐γ with agonists, such as thiazolidinediones (e.g., pioglitazone, troglitazone, and rosiglitazone), offers potential therapeutic benefits.^[^ ^13^ ^]^ One study confirming this concept was conducted by Fu et al., demonstrating that in situ eyelid injection of a rosiglitazone‐loaded hydrogel effectively delayed the MGs atrophy in the aging mice model.^[^ ^14^ ^]^

Contemporary approaches to managing MGD primarily rely on topical antibiotics, nonsteroidal anti‐inflammatory drugs, and steroids. However, conventional eye drops struggle to effectively reach MGs, due to rapid clearance and the conjunctiva barrier.^[^ ^15^ ^]^ While methods like subconjunctival or eyelid injections can be effective, they are associated with inherent drawbacks including pain, bleeding, infection risk, technical challenges, and limited patient acceptance.^[^ ^16^ ^]^ In contrast, microneedles (MN) technology offers a minimally invasive, controlled method for transdermal drug delivery. Widely used in vaccination, local anesthesia, and diabetic management, MN technology is known for its patient‐friendly design and efficacy.^[^ ^17^ ^]^ Despite its success in treating ocular diseases affecting the cornea, sclera, and retina,^[^ ^18^ ^]^ research on MN technology for eyelid‐related conditions like MGD remained relatively unexplored.

In this study, we developed a soluble, temperature‐responsive MN patch designed for transdermal drug delivery to the MGs. The patch was composed of poly(vinyl Alcohol) (PVA), cyclodextrin modified polyacrylic acid (PAA‐CD), and a novel indocyanine green derivative (IR820). PAA, a prototypical mucoadhesive polymer, is widely recognized for its strong bioadhesive properties,^[^ ^19^ ^]^ while CD is established carrier for delivering hydrophobic drugs.^[^ ^20^ ^]^ To enhance drug retention and enable sustained release following the dissolution of the MN, we selected PAA as the structure backbone and chemically linked CD through amide bonds. This innovative design allowed for the efficient loading and delivery of the PPAR‐γ agonist, rosiglitazone (ROSI). The formulation of the MN patch was optimized to ensure a safe temperature increase when exposed to near‐infrared ray (NIR) irradiation. Biocompatibility and safety assessments for eyelid applications were performed both in vitro and in vivo. The therapeutic efficacy of the ROSI‐loaded MN patch (ROSI‐MN) was evaluated in a high‐fat diet (HFD)‐induced MGD mouse model, showing improved outcomes compared to oral administration. Our results indicated that MN‐based transdermal drug delivery could provide a promising therapeutic option for treating eyelid‐related diseases such as MGD in the future.

## 2 Results and Discussions

### 2.1 Preparation of Photothermal MN Patches and Research Scheme

**Figure 1:**
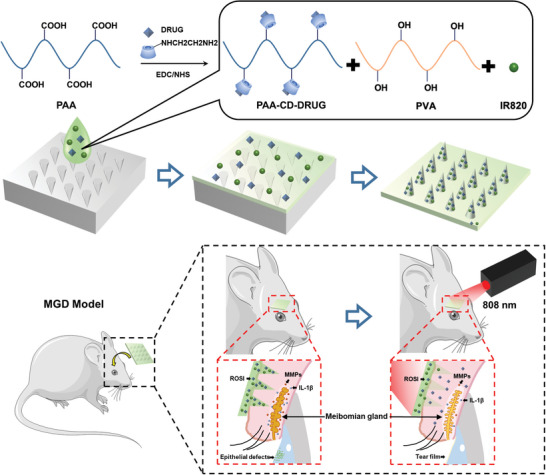
Schematic illustration depicting the fabrication and application process of the microneedle patch, alongside the mechanism of drug release from the microneedles to the meibomian glands under the illumination of an 808 nm laser, targeting the treatment of meibomian gland dysfunction in vivo.

MN has emerged as a highly effective and minimally invasive technique for transdermal drug delivery, facilitating the administration of small molecules and biomacromolecules into the skin with minimal discomfort and reduced invasiveness.^[^ ^21^ ^]^ This innovative approch has found board applications across diverse medical fields, including drug delivery for the skin,^[^ ^22^ ^]^ cardiac tissues,^[^ ^23^ ^]^ and cornea repair.^[^ ^18a,c^ ^]^ Given the sensitive nature of eyelid skin, MN technology presents a promising method for delivering drugs directly to MGs. To achieve controlled release of ROSI following MN insertion, PVA and PAA were selected as the base materials due to their exceptional biocompatibility, ease of molding, and strong tissue adhesion properties. The appropriate concentration of PVA and the optimized ratio of PVA to PAA could facilitate the removal of air bubbles from the mold, thereby ensuring the integrity of the MN tips. Additionally, this combination effectively modulated the dissolution rate of the MN. Further enhancing ROSI loading and release, PAA was modified with cyclodextrin (Figure S1, Supporting Information). Additionally, the system incorporated a photothermal agent, IR820 to facilitate the process. Following MN insertion, near‐infrared (NIR) irradiation (808 nm) was applied to accelerate MN dissolution and promote the release of ROSI to the MGs (**Figure** **1**). The NIR irradiation also generated localized heating, which assisted in the exclusion of meibomian lipids. The appropriate storage conditions for the MN patch depend on the specific components used. While PVA and PAA‐CD exhibit stability at room temperature, IR820, and ROSI require storage at 4 or −20 °C to maintain efficiency, with IR820 necessitating protection from light. Thus, the patch should be refrigerated at 4 °C in light‐proof packaging. For extended storage, the patch must be sealed and kept at −20 °C, with precautions taken to minimize air exposure before rewarming to prevent moisture absorption, which could impair tissue penetration efficacy.

### 2.2 Photothermal and Dissolution Behavior of MN Patches

**Figure 2:**
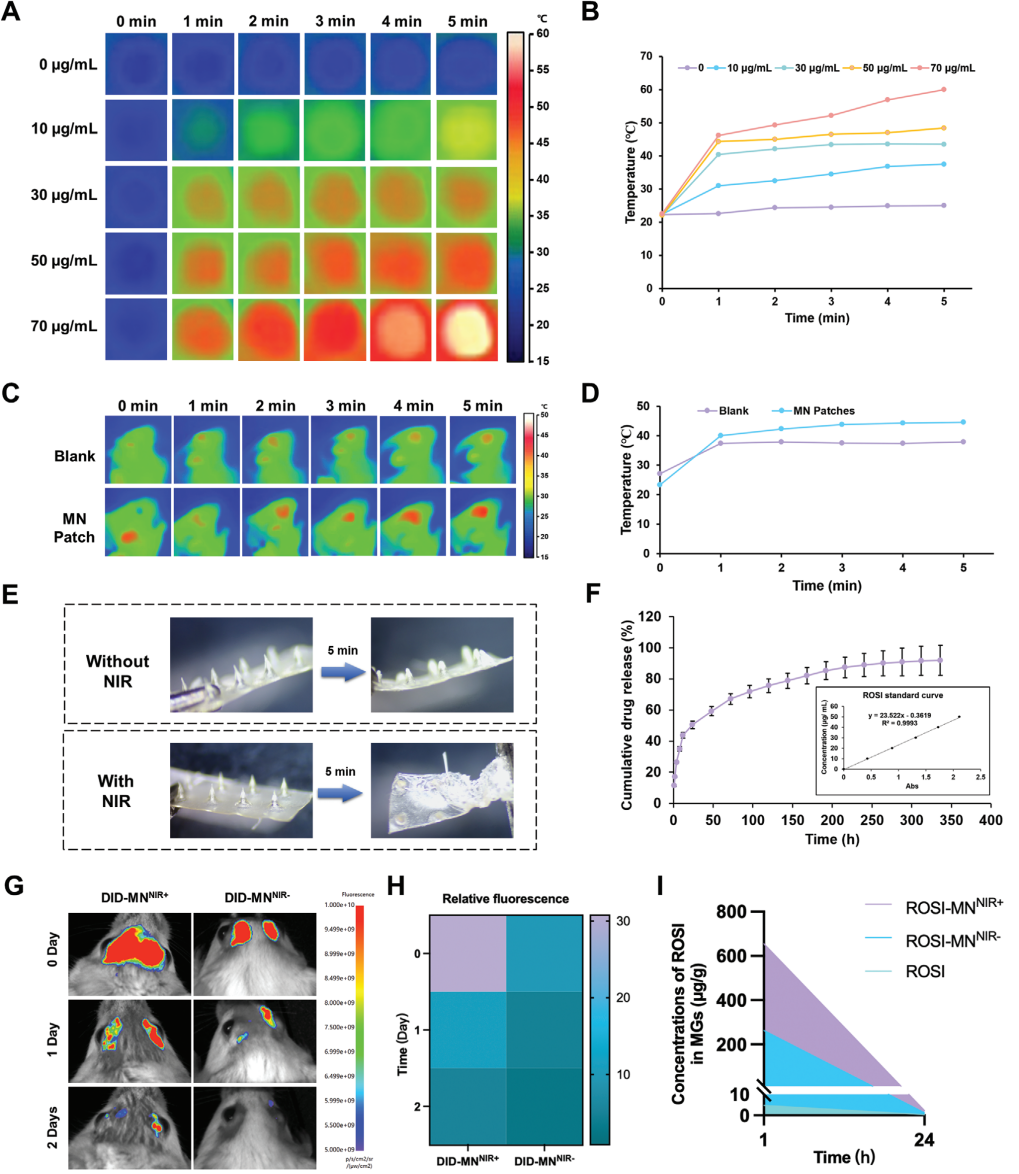
Photothermal and dissolution behavior of the MN patch with NIR irradiation. A) Real‐time infrared thermal images of the MN patch with gradient concentrations of IR820 in vitro. B) Temperature curves of the MN patch with gradient concentrations of IR820 in vitro. C) Real‐time infrared thermal image of the MN patch with 30 µg mL^−1^ IR820 in vivo. D) Temperature curve of the MN patch with 30 µg mL^−1^ IR820 in vivo. E) The representative images of the MN patch before and after insertion into murine eyelid without (left) and with (right) NIR irradiation. F) The release curve of ROSI from the MN patch. G) Representative IVIS images at specified time points after treatments with the CM‐DID loaded MN patch either with NIR irradiation or not. H) The relative fluorescence of CM‐DID was compared. I) Concentrations of the released ROSI in the murine MGs were compared at different timepoints post‐treatment.

The eyelid skin is exceptionally thin and densely innervated, making it highly sensitive to temperature changes. The LipiFlow system, commonly used in clinical settings for managing MGD, provides controlled heat at approximately 42.5 °C to the inner MGs, a temperature regarded as safe for eyelid warming.^[^ ^7^ ^]^ In our design, the heating rate and peak temperature of the MN patches were precisely controlled by adjusting the concentration of IR820. To evaluate the photothermal properties of the MN patches, we initially conducted in vitro assessments by varying IR820 concentrations (0‐70 µg mL^−1^). Infrared thermal imaging showed that IR820 enhanced the conversion of light into heat (**Figure** **2**A). As IR820 concentration increased, the heating rate and peak temperature of the MN patch also rose, stabilizing within 5 minutes (Figure 2A,B). At the concentration below 30 µg mL^−1^, the heating effect was insufficient to effectively facilitate MN dissolution. Notably, temperatures above 40 °C are essential for liquefying the obstructive glandular lipids that cause MGD.^[^ ^24^ ^]^ However, concentrations exceeding 30 µg mL^−1^ resulted in excessive temperatures, increasing the risk of discomfort or burns. Therefore, 30 µg mL^−1^ IR820 was identified as the optimal concentration for subsequent experiments, unless specified otherwise. The photothermal behavior of the MN patches was further assessed in vivo (Figure 2C,D). Upon insertion into the eyelid skin of mice and exposure to infrared irradiation, the MN patch exhibited a marked photothermal effect. The in vivo temperature profiles aligned with the in vitro results. However, technological limitations restricted temperature measurements to the skin surface, preventing direct assessment of temperature within the MN and MG tissues. Considering the penetration of infrared light and the thermal conductivity of biological tissues, it is plausible that the internal MN temperature was slightly lower than the recorded surface temperature.

Next, we investigated the dissolution behavior of the MN patches under infrared irradiation. The MN patch was inserted into a 1.5% agarose‐based simulated skin model and subjected to either infrared irradiation (With NIR) or no irradiation (Without NIR). It was found that a higher PVA ratio slightly increased the in vitro solubilization time (Figure S2, Supporting Information), likely due to the formation of hydrogen bonds between the additional hydroxyl groups on PVA and the carboxyl groups on PAA‐CD. NIR irradiation accelerated the dissolution of MN, as confirmed across all groups (Figure S2, Supporting Information). To achieve faster drug delivery into tissues while accommodating more PAA‐CD for enhanced drug loading, a PVA:PAA‐CD ratio of 50:17 was selected for subsequent experiments. To further simulate the diffusivity of the MN post‐dissolution in vitro, an orange dye was incorporated into the MN patches. The dissolution process and the diffusion of the dye were monitored over time, with and without NIR irradiation (Figure S3, Supporting Information). The results showed that the MN patches dissolved, and the orange dye gradually diffused into the agarose, with the process occurring more rapidly under NIR irradiation. However, the agarose hydrogels contained more water than in vivo tissues, likely contributed to faster dissolution. Consequently, the actual dissolution rate in biological tissues may be slower. To confirm this, we examined in vivo dissolution in murine eyelids (Figure 2E). After five minutes of insertion, part of the MN tips had dissolved. With NIR irradiation, the dissolution rate further increased, and the tips fully dissolved, remaining embedded within the tissue. These findings demonstrated that NIR irradiation enhanced the solubility of the MN.

Traditional eyedrop therapies show limited effectiveness for MGD due to the epithelial barrier and tear washout.^[^ ^25^ ^]^ Furthermore, conventional topical treatments and local injections often cause a burst drug release followed by a short duration of action, which is suboptimal for managing chronic progressive eye diseases.^[^ ^26^ ^]^ In contrast, MN technology offers significant advantages, including being painless, bloodless, and ease to administrate. Moreover, MN patches can act as drug reservoirs for controlled release.^[^ ^27^ ^]^ In our design, drug release from the MN patch followed a two‐stage mechanism: first, the MN dissolved, allowing ROSI‐infused solution to diffuse into the tissue, followed by the gradual release of ROSI from the solution or the CD cavities. The dissolution rate was modulated by the ratio of PVA to PAA‐CD, with the PAA‐CD content influencing both drug loading capacity and release profile. Although IR820 did not fundamentally alter drug release kinetics, it accelerated MN dissolution under NIR by raising local temperatures, thereby enhancing drug diffusion. To ensure thermal safety, we maintained a constant IR820 concentration. Following optimizing the PVA: PAA‐CD ratio and IR820 content, we investigated the sustained drug release duration to determine the optimal treatment frequency. *Ex vivo* analysis indicated that the MN patches provided gradual and sustained release of ROSI over the first 100 hours (Figure 2F). We further assessed sustained drug release in vivo using an IVIS (Figure 2G; Figure S4, Supporting Information). Fluorescent dyes (CM‐DID) loaded in the MN patch enabled us to monitor fluorescence signal changes, resealing insights into both hydrogel disassembly and drug release dynamics. Notably, residual fluorescence was detected in the eyelid area two days after a single MN treatment with NIR irradiation, confirming effective drug distribution and sustained drug release through the eyelid. By comparing fluorescence intensity, we found that NIR irradiation significantly enhanced the amount of drug released into the murine eyelids (Figure 2H). In addition, we evaluated the pharmacokinetics of ROSI using liquid chromatography‐tandem mass spectrometry (LC‐MS/MS) (Figure 2I). Following oral administration, the peak plasma concentration of ROSI typically occurs at approximately 0.75 hours (range: 0.75 to 1.0 hours).^[^ ^28^ ^]^ Our results showed that ROSI concentrations in MGs at 1 and 24 hours after the ROSI‐MN treatment, with or without NIR irradiation, significantly surpassed those achieved through oral administration.

The encapsulation efficiency of PAA‐CD was measured at 10.29 ± 0.21%, lower than that of CD alone, likely due to limitations in the grafting efficiency of CD onto PAA. Consequently, the fabricated MN contained a higher proportion of free ROSI and a lower concentration of ROSI encapsulated within the CD cavities. This unique composition enabled an initial burst release of ROSI, providing immediate therapeutic effects, followed by a sustained release to maintain localized drug levels within the tissue. Based on these results, we established a treatment frequency of once every 3 days for subsequent experiments. Although the frequent treatment was still required for MGD in this study, we anticipate that MN technology could revolutionize the long‐term management of chronic ocular diseases in the future.

### 2.3 Mechanical Behavior and Penetration Ability of MN Patches

**Figure 3:**
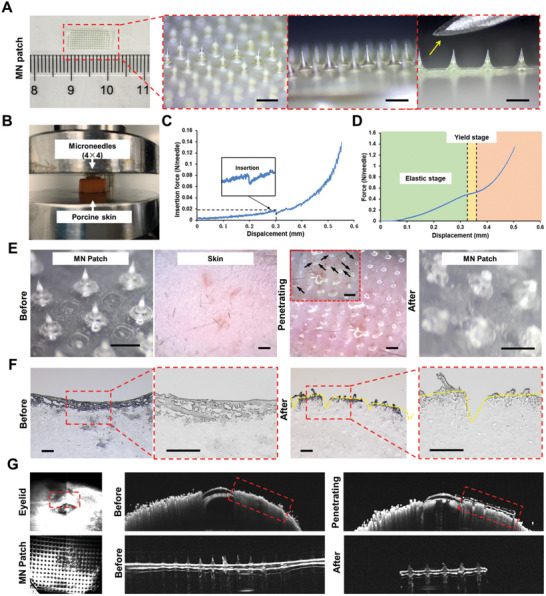
General view and mechanical properties of the MN Patch. A) Representative optical images and bright‐field microscopy images of MN patches and the 26 G needle head (yellow arrow) were shown. The red dashed box represented the enlarged image. Scale bar: 500 µm. B) The experimental setup of the mechanical testing device. C) Penetration behavior of the MN patch under compression using a vertical force. D) Mechanical behavior of the MN patch under compression using a vertical force. E) Representative bright‐field microscopy images of MN patches before, during, and after insertion into murine back skin. The red dashed box showed the enlarged image. The black arrows indicated the MN that inserted into the skin at higher magnification. Scale bar: 500 µm. F) Representative frozen section images of murine back skin before and after insertion. The red dashed box displayed specific enlarged areas. The yellow dashed lines highlighted the surface contour of the skin. Scale bar: 200 µm. G) Representative AS‐OCT images of the murine eyelid and the MN patch before, during, and after the insertion. The red dashed box showed the location of the shaved murine upper eyelid.

The MN micromold used in this study consisted of 385 conical needles, with a tip‐to‐tip spacing of 700 µm. Each needle cavity featured a base diameter of 270 µm and a height of 500 µm. After demolding and drying, the resulting MN patch (**Figure** **3**A) could be customized to meet specific sizes requirements. Microscopic analysis confirmed consistent needle tip morphology across different areas of the patch. Upon preparation, the size of the MN patch was slightly smaller than the original mold dimensions, with each needle measuring approximately 420 µm in height. This size reduction was likely due to volumetric contraction of PVA/PAA‐CD material during dehydration and drying. The needles were significantly smaller than that of a 26‐gauge (G) hypodermic needle, enabling non‐invasive and painless drug delivery.

A thorough evaluation of the MN patch's mechanical strength and penetration ability was performed (Figure 3B). Mechanical curve analysis revealed a distinct bending point, confirming successful penetration into porcine skin. Each individual needle required approximately 0.02 N of force for penetration (Figure 3C), while the entire MN patch withstand a maximum pressure of approximately 0.5 N (Figure 3D). Combined with the result of Figure 3C,D, our MN were proved to possess the sufficient strength. The force (0.5 N) exceeded the threshold required for MN to penetrate skin tissues (0.02 N), demonstrating its capability for efficient tissue penetration. As shown in Figure S5 (Supporting Information), MN insertion produced visible microchannels in agarose hydrogel, underscoring the importance of sufficient mechanical strength to prevent needle bending or fracturing during skin insertion. In vivo tests showed that the MN easily penetrated murine back skin, creating microchannels visible in frozen sections, although they were not clearly discernible to the naked eye (Figure 3E,F; Figure S6, Supporting Information). For enhanced visualization of MN penetration into the eyelid, anterior segment optical coherence tomography (AS‐OCT) imaging was employed, clearly depicting MN inserting into the shaved eyelid of a mouse (Figure 3G; Figure S7, Supporting Information). The tissue is tough, and the MN that is too short may fail to penetrate sufficiently to reach the desired depth. As shown in Figure 3G, the MN did not perforate the entire eyelid, indicating that this length was appropriate. Thus, the MN length of the mold needs to account for factors such as volume changes during preparation, as well as the thickness and hardness of the target tissue. For applications on thicker animal eyelids, longer MN should be prepared to ensure adequate penetration into deeper layers. In addition, the prepared MN featured a soft base layer, determined by its primary composition. PVA exhibited excellent film‐forming properties, enabling the dried film to bend easily without breaking. Moreover, the combination of PVA and PAA provided strong tissue adhesion, reducing the likelihood of displacement after application. This facilitated secure application and ensured effective penetration. These results confirmed that the MN patch possessed adequate mechanical strength for tissue penetration, supporting its suitability for subsequent drug delivery and therapeutic applications.

### 2.4 Biocompatibility and Biosafety of the MN Treatment

**Figure 4:**
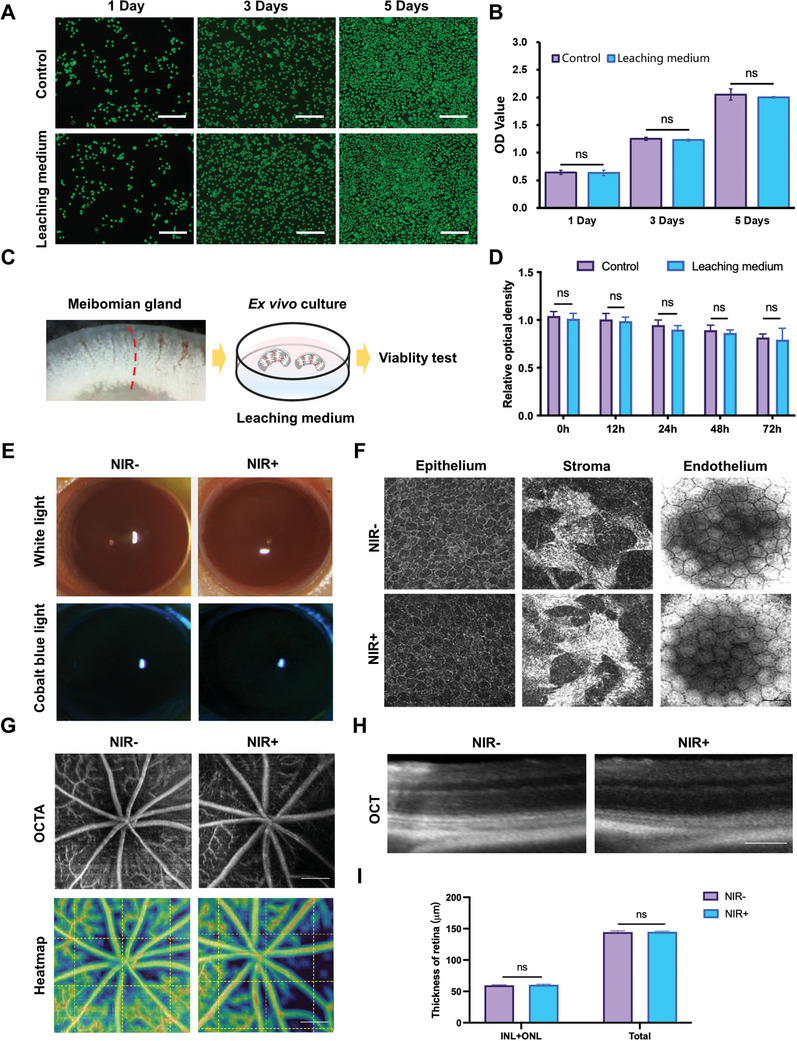
Biosafety of the MN patch and NIR laser irradiation. A) Representative Live&Dead images of L929 cells cultured in complete medium (Control) and leaching medium for 1, 3, and 5 days, respectively. Scale bar: 100 µm. B) CCK‐8 assays of L929 cells cultured in complete medium and leaching medium on Day 1, 3, and 5. C) The schematic representation of MGs tissue culture model. The MG explants were carefully transferred to a 24‐well culture plate and merged with either complete medium (Control) or leaching medium. D) Relative optical densities of MGs after culture were compared. E) Representative bright field (upper) and fluorescein staining (lower) images depicting corneal opacity and integrity of corneal epithelium in NIR‐ and NIR+ groups. F) Representative images of FF‐OCT to measure the corneal cellular morphology and density at different layers. Scale bar: 25 µm. G) Representative OCTA (upper) and heatmap (lower) images of fundus manifesting central retina vessels from two groups. Scale bar: 100 µm. H) Representative OCT images of transverse sections of retinas from two groups. Scale bar: 100 µm. I) Thicknesses of INL and ONL, as well as total retinas were compared. Two‐way ANOVA was performed for comparisons among groups. ns: *p* > 0.05.

The biosafety of the MN materials for transdermal drug delivery was initially assessed using L929 cells. Live&Dead staining showed no significant difference in cell viability between cells cultured in the leachate from the MN patch, composed of PVA, PAA‐CD and IR820, and those cultured in the complete medium (**Figure** **4**A). Moreover, the CCK‐8 assay indicated that exposure to the leachate did not impair the proliferative capacity of L929 cells at 1, 3, and 5 days post‐treatment (Figure 4B). Further biosafety evaluation for drug delivery to the MGs were performed using ex vivo murine MG explants, followed by CCK‐8 assays. Organotypic cultures of MG explants, which retained significant metabolic activity, provided a reliable alternative for in vivo biosafety evaluations targeting MGs.^[^ ^29^ ^]^ As shown in Figure 4C, MG tissue viability in the leachate was comparable to that in the complete medium (Figure 4D), indicating no significant adverse effects. These results suggested that the MN patch materials exhibit minimal toxicity to MG tissues, supporting their potential for further applications.

Excessive light exposure can damage tissues through photothermal, photomechanical, and photochemical mechanisms.^[^ ^30^ ^]^ In this study, we used NIR irradiation (808 nm) to accelerate MN dissolution and drug release. The safety of NIR irradiation for the cornea has been demonstrated in previous studies, where NIR‐triggered hydrogel adhesives were applied to corneal injuries without causing harm to corneal cells.^[^ ^31^ ^]^ Furthermore, numerous studies have shown that NIR light promoted wound healing, reduced retinal damage, and supported nerve repair.^[^ ^31^, ^32^ ^]^ To evaluate the phototoxicity of NIR within the applied intensity, we administrated NIR irradiation every three days for 5 minutes over 3 months. As shown in Figure 4E and Figure S8A (Supporting Information), no corneal opacity or epithelial defects were detected under slit‐lamp microscope. Similarly, full‐field optical coherence tomography (FF‐OCT) images revealed no abnormalities in the morphology or density of the corneal epithelium, stroma, or endothelium (Figure 4F; Figure S8B, Supporting Information). These findings align with previous study reporting no adverse effects on corneal cells from direct irradiation during hydrogel‐based treatment.^[^ ^31a^ ^]^ Generally, wavelengths ranging from 760 to 1400 nm pass through the cornea with minimal absorption, reaching the retina, which is considered the primary site for potential phototoxicity.^[^ ^33^ ^]^ To assess this, we examined the retina using optical coherence tomography angiography (OCTA), which showed no differences between the NIR‐treated and untreated groups (Figure 4G; Figure S8C, Supporting Information). Further assessment of retinal thickness and microstructure through OCT revealed no significant differences in the combined thickness of the inner nuclear layer (INL) and outer nuclear layer (ONL), or in total retinal thickness, between the two groups (Figure 4H,I; Figure S8D, Supporting Information). Collectively, these results indicated that the NIR irradiation applied in this study did not induce ocular side effects during MGD treatment.

The biosafety of the MN patch for MGs was further evaluated in vivo. A blank MN patch, with a 5 × 1‐needle configuration covering an area of 3.5 mm × 0.7 mm, was applied to the upper eyelid skin of mice every 3 days for 3 months. Representative histological images of cross‐sectioned MGs at various time points are shown in Figure S9 (Supporting Information). The microstructure of MGs in the MN‐treated group remained consistent with that of normal glands. Additionally, no signs of inflammatory cell infiltration or fibrotic damage was observed after 3 months of repeated MN applications. These findings, obtained from both *ex vivo* and in vivo biosafety evaluations at the cellular and tissue level, demonstrated that MN application to the eyelids was safe and suitable for MGD treatment.

### 2.5 Therapeutic Effects of ROSI‐Loaded MN Patch on MGD In Vivo

The MGs are specialized sebaceous glands in the eyelids responsible for producing meibum, a vital tear film component. Meibum plays a key role in maintaining ocular surface homeostasis by stabilizing the tear film and reducing evaporation.^[^ ^4^ ^]^ Currently, MGD has reached epidemic proportions, affecting approximately 46.2% to 69.3% of the global population.^[^ ^1^, ^2^ ^]^ Previous clinical studies have suggested that dyslipidemia, particularly that associated with obesity, might alter the lipid composition of meibum, contributing to an increased risk for MGD.^[^ ^34^ ^]^ Animal experiments have demonstrated that dyslipidemia could alter meibum lipid composition, leading to inflammatory events within MGs, thereby perpetuating the vicious circle of MGD and ocular surface damage. Mice fed a HFD exhibited clinical signs and pathological changes consistent with MGD and dry eye‐like injuries,^[^ ^12^, ^35^ ^]^ making them a reliable model for further study. PPAR‐γ is a critical regulator of meibocyte differentiation and lipid synthesis.^[^ ^10^ ^]^ According to Bu et al., excessive lipid accumulation in acinar cells downregulated PPAR‐γ expression, contributing to aberrant MG morphogenesis and increased inflammatory cell infiltration.^[^ ^10^, ^36^ ^]^ Based on this, we selected ROSI, a PPAR‐γ agonist, for transdermal delivery via the MN to treat MGD. The experimental setup included the ROSI‐MN group, a blank MN patch control (MN group), and systemic administration of ROSI (ROSI group).

**Figure 5:**
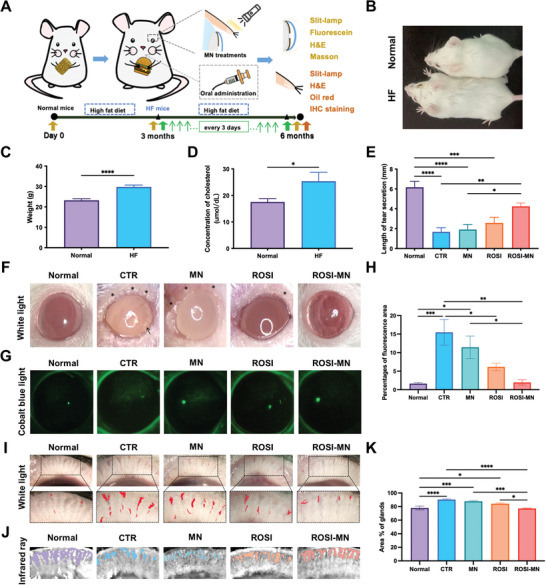
Therapeutic effects of ROSI‐MN application on the HFD related abnormalities of MGs. A) Schematic for the animal experimental design to illustrate therapeutic effects of ROSI‐MN in the HFD induced MGD murine models. B) The body sizes of mice fed a SFD (Normal) or HFD (HF) for 12 weeks. C,D) Body weights (C) and total cholesterol concentrations (D) of normal and HF mice were compared. E) Measurements of tear secretions using the phenol red thread in mice. F) Representative gross images of ocular surface showed the morphology of eyelid margins and corneas in mice. Black stars indicated areas with obvious anomalies and the black arrow pointed toward new blood vessels on the cornea. G) Representative fluorescein staining images of corneas demonstrating the epithelial defects in each group. H) Percentages of corneal staining areas from each group were compared. I) Representative in vivo bright field images of MGs. The black dashed line represented enlarged views of central areas and the red areas highlighted capillaries. J) Representative infrared images of MGs ex vivo. Colored areas represented the gaps between meibomian glands. K) Comparison of percentages of glandular areas in eyelids. Student's *t*‐test was performed for the comparison between two groups and one‐way ANOVA was performed for the comparison among five groups. *p < 0.05, **p < 0.01, ***p < 0.001, ****p < 0.0001.

As illustrated in **Figure** **5**A, mice underwent gross examinations before sacrifice to allow for further morphological and molecular evaluations. After the 3‐month feeding period, the high fat dieted (HF) mice weighed approximately 28.2% more than the normal mice. This weight gain was accompanied by elevated plasma cholesterol levels compared to those on the standard fat diet (SFD) (Figure 5B–D). Given that MGD is a major cause of dry eye, tear volume was evaluated using the phenol red cotton thread test. Consistent with previous findings, tear secretion was significantly reduced in HF mice of the CTR and MN groups, indicating successful induction of the MGD model (Figure 5E).^[^ ^35^ ^]^

Both systemic administration and MN delivery of ROSI increased tear secretion, with the ROSI‐MN group showing the most significant improvement. Additionally, the CTR, MN, and ROSI groups exhibited milky secretions along the hypertrophic eyelid margins, and corneal neovascularization was even observed in the CTR group. In contrast, the eyelid margins of the ROSI‐MN treated mice resembled those of normal mice, displaying minimal milky secretion and smooth eyelid surfaces (Figure 5F; Figure S10A, Supporting Information). These findings indicated that MGD could severely affect ocular surface health.^[^ ^4^, ^37^ ^]^ Notably, ROSI‐MN treatment protected the ocular surface in the MGD mice, resulting in a significant reduction of corneal epithelial defects. As shown in Figure 5G and Figure S10B (Supporting Information), the corneas of mice in the CTR and MN groups exhibited severe fluorescein staining, demonstrating that HFD disrupted ocular surface integrity through the successful MGD induction. However, corneal staining was markedly reduced in both the ROSI and ROSI‐MN group (Figure 5G; Figure S10B, Supporting Information). Quantitative analysis of corneal epithelial defects indicated that slight improvement in the MN group, possibly due to the heat from NIR irradiation. ROSI oral administration had significant therapeutic effects, but the ROSI‐MN group provided the most effective protection against MGD‐related injuries (Figure 5H). These results demonstrated that HFD could successfully induce MGD‐related injuries and suggested that ROSI‐MN treatment effectively restored ocular surface homeostasis.

In addition to in vivo slit lamp examinations, we isolated MGs for *ex vivo* infrared imaging to quantitatively analyze morphological changes. Both white light and infrared images showed larger MG areas in their eyelids of HFD‐fed mice compared to normal mice, with increased gland width thought to precede MGD and exacerbate ocular surface damage (Figure 5I,J; Figure S10C,D, Supporting Information).^[^ ^34b^ ^]^ Although MGs length did not significantly differ between groups, inflammation, such as eyelid hyperemia (indicated by the red areas), were more pronounced in the HF mice (Figure 5I; Figure S10C, Supporting Information). These morphological abnormalities, characteristic of MGD, were alleviated by ROSI‐MN treatment (Figure 5I–K). ROSI oral administration also improved MG morphology (Figure 5I–K),^[^ ^12^, ^14^ ^]^ but the oral dose (300 µg every 3 days) was approximately 15 times higher than that delivered via the MN patch (around 20 µg every 3 days). Thus, ROSI‐MN treatment reduced costs and minimized systemic effects.

Additionally, MN group showed slight decreases in corneal fluorescein staining, congestion, and MG enlargement compared to the CTR group, likely attributable to the photothermal effect. However, these findings highlighted the limitations of heat alone as a therapeutic measure, as its effects were far inferior to those of ROSI‐MN treatment. Overall, ROSI‐MN treatment showed great potential in treating MGD‐related ocular surface damages caused by high‐fat intake. Future studies should apply this approach to other models of MG‐related disease, such as age‐related MGD and hyperglycemia‐induced MGD, to further validate the therapeutic efficiency of ROSI‐MN treatment. For clinical translation, comparisons between current practices, such as manual compression or the LipiFlow system, and the drug loaded MN patch should be explored to pave the way for comprehensive comparisons of these approach.

**Figure 6:**
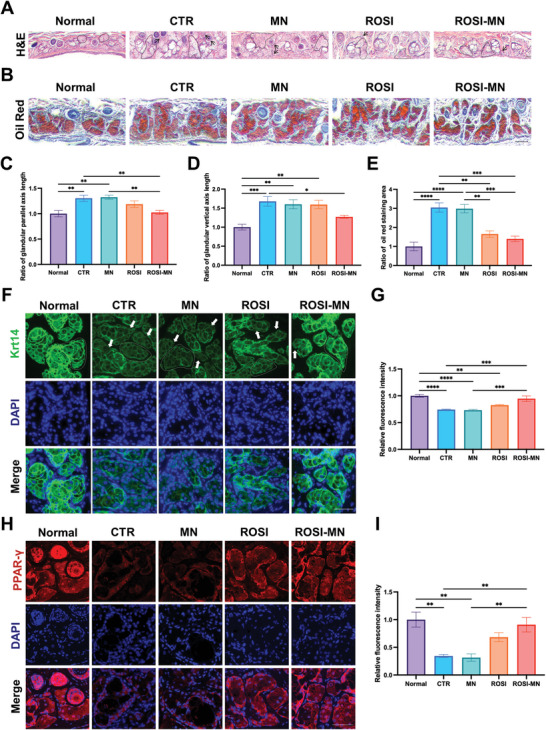
ROSI‐MN treatment improved the recovery of MGs via increasing PPAR‐γ expression. A) Representative H&E staining showed the microstructure of MG acinus from each group after 3‐month treatments. The black arrows pointed toward monocytes and the dotted line protruded the shapes of acinus. Scale bar: 50 µm. B) Representative Oil Red O staining images of MGs indicating lipid areas of MGs in each group. Scale bar: 50 µm. C,D) The lengths of both parallel axis (C) and perpendicular axis (D) to the conjunctival surface showed decreased sizes of acinus after treatments. E) Statistics of Oil Red O staining areas from MGs of each group. F) Representative Krt14 (green) staining images of MGs. White arrows showed the destroyed acini membrane and the dotted white line protruded the shapes of acinus. Scale bar: 50 µm. DAPI stained nuclei (blue). G) Relative fluorescent intensity of Krt4 in MGs from each group. H) Representative immunofluorescent staining images of PPAR‐γ (red) in the MGs. Scale bars: 50 µm. DAPI stained nuclei (blue). I) Relative fluorescent intensity of PPAR‐γ in MGs from each group. One‐way ANOVA was performed for the comparison among five groups. *p < 0.05, **p < 0.01, ***p < 0.001, ****p < 0.0001.

To further assess MG morphology, H&E, and Oil Red O staining were performed on sectioned eyelid tissues after treatment. In H&E images, the MG acini of normal mice exhibited a spherical, foam‐like appearance, while those in HF mice appeared irregular and disorganized. Following treatment, the MG acini in both the ROSI and ROSI‐MN groups closely resembled those in normal mice (**Figure** **6**A; Figure S11A, Supporting Information). Additionally, the number of monocytes surrounding the acini, indicative of inflammation (black arrows), was significantly reduced in both the ROSI and ROSI‐MN groups compared to untreated HF mice (CTR), suggesting reduced inflammation. Oil Red O staining revealed substantial lipid droplet accumulation in the dilated acini of HF mice (Figure 6B; Figure S11B, Supporting Information). Further analysis of acini axis lengths showed that ROSI‐MN treatment significantly limited MG enlargement (Figure 6C,D). Moreover, both oral ROSI administration and ROSI‐MN treatment effectively reduced lipid accumulation in MGs, with ROSI‐MN treatment showing a more pronounced effect (Figure 6E). Keratin (Krt) 14, a marker for all stratified epithelia, including the acinus and the ducts of MGs, is crucial for maintaining physical stability.^[^ ^29^ ^]^ Immunofluorescence staining revealed a reduction Krt14 expression in MGs following HFD intake, whereas, ROSI‐MN treatment significantly restored Krt14 expression and preserved acini integrity (Figure 6F,G; Figure S11C, Supporting Information). Overall, both visual and histological analyses demonstrated that ROSI‐MN treatment effectively mitigated lipid metabolism disorder‐induced damage to MG structure.

Previous studies have indicated that HFD downregulated PPAR‐γ expression, leading to abnormal meibocyte differentiation and increased lipogenesis. In contrast, ROSI, a specific PPAR‐γ agonist, reversed this reduction, decreasing lipid accumulation in MGs.^[^ ^12a^ ^]^ Therefore, we examined PPAR‐γ expression in MGs across five groups and found that its fluorescence intensity in the control (CTR) group was significantly lower than that in normal mice, while ROSI‐MN treatment markedly improved PPAR‐γ expression compared to other treatments (Figure 6H,I; Figure S11D, Supporting Information). This finding was confirmed by western blot (WB) analysis, which showed reduced PPAR‐γ expression after prolonged HFD feeding (Figure S12, Supporting Information). The increased PPAR‐γ expression following ROSI‐MN treatment, in conduction with near‐infrared (NIR) light, also confirmed that this method preserved the pharmacological activity of ROSI. In conclusion, ROSI‐MN treatment demonstrated superior drug availability compared to traditional oral medications and offered greater convenience for self‐administration than previously reported eyelid subcutaneous injections, thus enhancing patient compliance and reducing associated risks.

**Figure 7:**
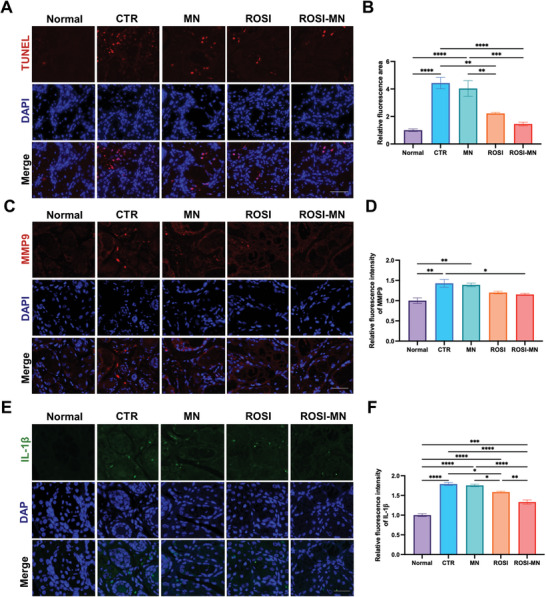
ROSI‐MN treatment reduced the HFD‐induced inflammation injuries in MGs. A) Representative images of TUNEL (red) staining in MGs. The cell nuclei were stained blue with DAPI. Scale bar: 50 µm. B) Relative fluorescent areas of TUNEL^+^ cells in MGs. C) Representative images depicting MMP9 (red) immunofluorescent staining in MGs. DAPI (blue) was used for nuclear staining. Scale bar: 50 µm. D) Comparison of the relative fluorescent intensity of MMP9 staining. E) Representative IL‐1β (green) immunofluorescent staining images of MGs. DAPI (blue) was used for nuclear staining. Scale bar: 50 µm. F) The relative fluorescent intensity of IL‐1β staining from each group were compared. One‐way ANOVA was performed for the comparison among five groups. *p < 0.05, **p < 0.01, ***p < 0.001, ****p < 0.0001.

To investigate the mechanism underlying the alleviation of MGs injuries, we performed TUNEL assay on MG tissues to identify apoptotic cells. While TUNEL positive cells were rare in the normal mice, they were evident in HF mice (**Figure** **7**A; Figure S13A, Supporting Information). Quantification of TUNEL positive cells further demonstrated that the HFD intake promoted MG acinar cell apoptosis, whereas ROSI‐MN treatment provided better protective effect than ROSI oral administration (Figure 7B). Previous studies have suggested that HFD‐related inflammation contributed to MG cell death, characterized by elevated levels of proinflammatory cytokines.^[^ ^12^, ^38^ ^]^ Therefore, we conducted immunofluorescent staining for IL‐1β and MMP‐9 in MG tissues. Our results showed significantly higher expression of both key factors in HF mice compared to normal ones, confirming the successful establishment of MGD model in our study (Figure 7C–F; Figure S13B,C, Supporting Information). Additionally, both oral ROSI administration and localized ROSI‐MN treatment significantly reduced HFD‐related inflammatory infiltration, with ROSI‐MN showing superior efficacy compared to orally ROSI (Figure 7C–F; Figure S13B,C, Supporting Information).

PPAR‐γ agonists are recognized as mediators of the ocular surface disease, such as dry eye and MGD, and have been shown to reduce the expression levels of IL‐1β and inhibit MMP‐9 activity.^[^ ^14^, ^39^ ^]^ Localized eyelid administration of ROSI, with sustained release, was more effective in promoting MG cell survival and reducing inflammation than oral therapy (Figure 7), suggesting that higher local ROSI concentrations in eyelids improved drug availability compared to traditional oral administration. Moreover, there was a trend toward reduced apoptosis in the MN group, which might be attributed to the photothermal effects that mitigated dyslipidemia and HFD‐induced changes in meibum lipids. Further investigations are needed to explore the specific contribution of thermal therapy in the ROSI‐MN treatment.

**Figure 8:**
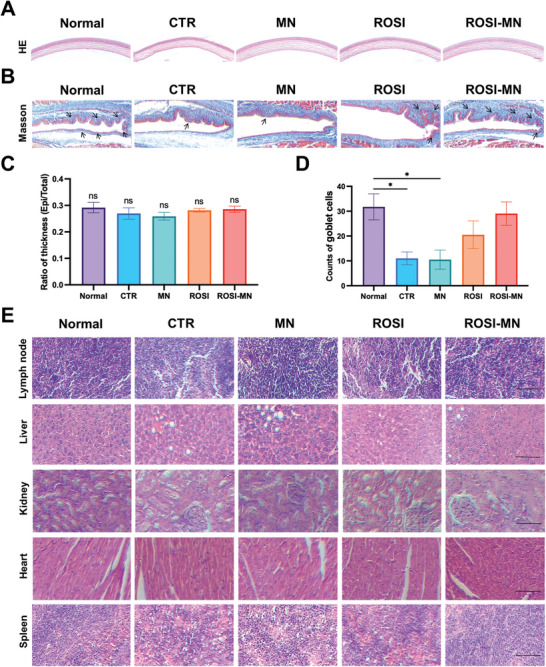
Biosafety analysis of ROSI‐MN treatment. A) Representative images of H&E staining of corneas. Scale bar: 50 µm. B) Representative Masson's trichrome images of the conjunctiva. Black arrows indicated the goblet cells. Scale bar: 50 µm. C) Ratios of corneal thickness were statistically analyzed. D) Comparison of goblet cell numbers in conjunctival fornix from each group. E) Representative images of H&E staining of cervical lymph node, liver, kidney, heart, and spleen from five groups. Scale bar: 50 µm. One‐way ANOVA was performed for the comparison among five groups. ns: *p* > 0.05, *p < 0.05.

We conducted a comprehensive investigation into the biosafety of the ROSI‐MN treatment to further evaluate its potential for clinical application. In the H&E stained corneal images, the only notable finding was a relatively irregular arrangement of collagen fibers in the corneas of the CTR group. In contrast, the corneas of mice in the treated groups displayed no discernible differences (**Figure** **8**A; Figure S14A, Supporting Information). Additionally, Masson's trichrome staining of the conjunctiva revealed that the fornices of all treated mice exhibited a structure comparable to that of normal mice (Figure 8B; Figure S14B, Supporting Information). Statistical analysis showed no significant differences in corneal thickness ratios among the various groups (Figure 8C).

However, we observed a significant reduction in goblet cell numbers in the CTR and MN group compared to normal mice (Figure 8D). Notably, treatments with orally administered ROSI and ROSI‐MN partially restored goblet cell counts. Moreover, there were no signs of inflammatory infiltration in the cervical lymph nodes, and the major organs—including the liver, kidneys, heart, and spleen—showed no significant changes in ROSI‐MN treated mice compared to the normal group (Figure 8E; Figure S14C, Supporting Information). These results suggested that ROSI‐MN treatment did not induce significant ocular surface and systemic side effects in the HFD induced MGD mice. Given the efficiency and safety of the drug‐loaded MN delivery system, it holds promise for other types of MGs diseases, such as those related to aging or infection.

## 3 Conclusion

MGD is a common ocular disorder that plays a major role in the development of dry eye diseases by disrupting the tear film and impairing ocular surface homeostasis. In this study, we developed a soluble, temperature‐responsive MN patch for transdermal drug delivery to the MGs. This noval MN design exhibited sufficient mechanical strength to penetrate the eyelid skin effectively. Upon NIR irradiation, the MN patch dissolved rapidly, enabling sustained drug release after insertion. Furthermore, leveraging its photothermal conversion properties, the MN patch also acted as a heating device, liquefying meibomian lipids and relieving duct obstructions. The transdermal drug delivery potential of this MN patch was validated in HFD mice, a well‐established model for MGD. Our results demonstrated that the MN patch overcame challenges related to drug permeation and targeted administration to the MGs, offering superior therapeutic efficacy compared to conventional oral drug delivery methods. In conclusion, this study highlighted the MN patch as a promising approach for targeted drug delivery to the eyelids, with potential applications of treating various ocular surface diseases.

## 4 Experimental Section

### Animals

All animal involved experiments were conducted in adherence to the experimental animal ethical guidelines for ophthalmic research provided by the Association for Research in Vision and Ophthalmology (ARVO) and were approved by the Medical Ethics Committee of Zhongshan Ophthalmic Center, Sun Yat‐sen University. Six week‐old male BALB/c mice were obtained from the Animal Center of Zhongshan Ophthalmic Center, Sun Yat‐sen University, and were housed in a standard pathogen‐free environment with a 12‐hour light/dark cycle.

### Synthesis of Cyclodextrin Modified Polyacrylic Acid (PAA‐CD)

PAA‐CD was synthesized using a previously reported method with certain modifications.^[^ ^40^ ^]^ The molecular weight (MW) of PAA is critical in influencing both the mechanical strength and dissolution rate of MN. Lower molecular weights improve dissolution effectively but limit the ability of the MN to penetrate tissue effectively, while higher molecular weights complicate preparation due to increased viscosity, which hinders bubble removal during molding and results in incomplete or missing MN. Therefore, 500 mg of PAA (MW approximately 250000, Sigma‐Aldrich, USA) was dissolved in 30 mL of deionized water. Then, 150 mg of 1‐Ethyl‐3‐(3‐dimethylaminopropyl) carbodiimide (EDC) (TCI, Japan) and 150 mg of N‐hydroxysuccinimide (NHS) (TCI, Japan) were added to the solution. Subsequently, 100 mg of mono‐(6‐ethanediamine‐6‐deoxy)‐β‐CD (Shandong Binzhou Zhiyuan Biotechnology Co., Ltd., China) was introduced. The reaction mixture was stirred continuously at room temperature overnight. Afterward, the mixture was dialyzed (MWCO: 14 kDa, Guangzhou Qiyun Biotechnology Co., Ltd., China) against deionized water for 72 hours to remove unreacted substances, followed by freeze‐drying to obtain the PAA‐CD.

### Fabrication of the MN Patch

MN patch was fabricated using a micromold method. First, PVA with 87∼89% degree of alcoholysis and 1700 degree of polymerization (Rhawn, China) was dissolved into deionized water to create a 5% solution. Then, PAA‐CD and IR820 (Shanghai Bide Pharmaceutical Technology Co., Ltd., China) were added to the PVA solution to reach a concentration of 1.7% and a gradient concentration of 0–70 µg mL^−1^, respectively. ROSI (MedChem Express, USA) or CM‐DID (Invitrogen, USA) was added to the mixture at a concentration of 2% to create a suspension. After 24 hours of stirring, the pre‐solution of MN was cast into a round polydimethylsiloxane (PDMS) female mold with a diameter of 17.5 mm (Suzhou Intelligent Manufacturing Research Institute, China). The micromold consisted of 385 conical needles, each with a tip‐to‐tip spacing of 700 µm. The needle cavities had a base diameter of 270 µm and a height of 500 µm. After air bubble removal via negative pressure, the micromold was left to dry overnight in a well‐ventilated area. The drug‐free (MN), ROSI loaded (ROSI‐MN) or CM‐DID loaded MN patch was then carefully peeled off from the PDMS mold and stored at −20 °C for future use.

### NIR‐Responsiveness of the MN Patch

The photothermal effect of MN patch was assessed both in vivo and vitro. Initially, the MN patches with varing concentrations of IR820 (0, 10, 30, 50 and 70 µg mL^−1^) were irradiated with NIR laser (808 nm, 1 W/cm^2^, model FU808AD1000‐F34, Shenzhen Fuzhe Technology Co., Ltd., China) for 5 min. Temperatures of MN patches were recorded at 1‐minute intervals using an infrared thermal camera (H11pro, Hikmicro, China). The MN patch containing 30 µg mL^−1^ IR820 reached a temperature of approximately 40 °C under NIR irradiation, a temperature closely matching the melting point of meibum lipids in MGD. Therefore, the MN patch with 30 µg mL^−1^ IR820 was selected for subsequent evaluations. Photothermal conversion properties of the MN patch were further evaluated on the eyelid skin of BALB/c mice in vivo. First, the MN patch (4 × 2 needles) was applied to shaved murine eyelid skin by pressing for 5 s and left in place. Next, the MN patch was exposed to the NIR irradiation for 5 min. Thermal images and temperature curves were recorded. Mice exposed to NIR irradiation without the MN patch served as the Blank group.

### Dissolution Properties of the MN Patch

The dissolution properties of the MN patch were initially tested using a 1.5% (w/v) agarose gel (Biowest, Spain) as a model for skin. The MN patch was pressed against the gel and removed after varying durations (10, 30, and 60 sec), with or without NIR irradiation. The morphological changes of MNs and the microchannels created in agarose gel were examined using a stereoscopic microscope (Axio Imager Z2, Zeiss, Germany). Dissolution of the MN patch was also assessed in vivo. The MN patch (4 × 2 needles) was inserted into the shaved murine eyelid skin for 5 s and left in place for 5 minutes, without NIR or with NIR. The MN patch morphology images before and after insertion were compared using a stereoscopic microscope.

### Encapsulation Efficiency and In Vitro Drug Release Assay

The encapsulation efficiency and release profile of ROSI from the ROSI‐MN patch was determined using a UV3802 ultraviolet visible spectrophotometer (Shanghai UNICO, China). As reported in previous study, the optimum detection wavelength for ROSI is 245 nm.^[^ ^14^ ^]^ Initially, a series of standard samples with different concentrations (0, 10, 20, 30, 40, and 50 µg mL^−1^) were prepared to establish a standard curves of absorbance. To measure encapsulation efficiency, PAA‐CD was dissolved in deionized water, followed by the addition of ROSI to create a suspension. The mass ratio of PAA‐CD to ROSI was maintained at 17:20, consistent with the MN patch formulation. After 24 hours of stirring, the mixture was centrifuged for 10 minutes at 4000 rpm min^−1^ to obtain the supernatant. The supernatant's absorbance was measured, and the encapsulation efficiency was calculated based on the standard curve. The drug‐loaded MN patch was then immersed in 1 L of a receiving solution (PBS, PH 7.4) with gently agitation (60 rpm min^−1^) in a heating cabinet at 37 °C for dialysis. The receiving solution was collected at specific intervals (1, 2, 4, 8, 12, 24, 48, 72, 96, 120, 144, 168, 192, 216, 240, 264, 288, 312, and 336 hours), and analyzed for drug concentrations by measuring absorbance. All experiments were performed in triplicate.

### In Vivo Drug Release Assay

An in vivo fluorescence imaging system (IVIS) was utilized to evaluate the drug release profiles of MNs in murine eyelid. A CM‐DID loaded MN patch (DID‐MN, 5 × 1 needles) was applied to the shaved murine eyelid skin, with the patch being pressed for 5 s and left in place for 5 minutes, either with NIR (DID‐MN^NIR+^) or without NIR (DID‐MN^NIR−^) irradiation. IVIS was then employed to measure the remaining dye in vivo on Day 0, Day 1, and Day 2 post‐application. Mice that did not receive MN treatment were designed as the Control group. All images were captured under the same exposure setting, and fluorescence intensities from the uniform‐sized areas on each MG were calculated.

### Detection of ROSI Content In Vivo

The in vivo ROSI concentrations within MGs were accurately measured using liquid chromatography‐mass spectrometry (LC‐MS/MS, Thermo Fisher Scientific Inc., Boston, USA). Normal mice were treated either with ROSI‐loaded MNs followed by NIR irradiation (ROSI‐MN^NIR+^), ROSI‐loaded MN without NIR (ROSI‐MN^NIR−^), or administered orally of ROSI at 10 mg kg^−1^. The ROSI‐MN patches (5 × 1 needles) and NIR were applied as previously described, and MGs (n = 3 per group) were harvested at 1 hour and 24 hours post‐treatment. After weighing, six times the sample mass in methanol was added, and samples were homogenized for 1 minute, followed by vortexing and thorough mixing. The samples were then centrifugated at 10 000 rpm for 10 minutes, and the supernatants were collected for LC‐MS/MS analysis.

### Mechanical Properties of the MN Patch

The morphology of the MN patch was observed using a mobile phone camera and a stereoscopic microscope. The mechanical strength of the MN patch was assessed with a universal material testing machine (CMT‐1104, Zhuhai SUST Electrical Equipment Co., Ltd., China). The MN patch (4 × 4 needles) was affixed on a rigid stainless‐steel plate with the needles facing upward. Ex vivo porcine skin tissue or a smooth stainless‐steel sheet was placed against a cylindrical probe, which moved vertically toward the MN patch at a constant speed of 100 µm s^−1^. The variation in needle compression force with the probe displacement was recorded, and the insertion force was determined at the point where a force discontinuity followed by a steep rise, was observed.

### Skin Insertion Tests In Vitro and In Vivo

The penetration capability of the MN patch was tested both in vitro and in vivo. Initially, a properly sized MN patch was applied to 1% agarose gel in vitro. The base layer of the MN patch was attached to a flat metal sheet that was moved vertically toward the gel. The morphological changes in both the MNs and the agarose gel before and after insertion were examined using a stereoscopic microscope. To further assess penetration ability in vivo, the MN patch was applied to shaved dorsal and eyelid skin of mice for 5 s. After removal, the MN patch and the treated area were examined before, during, and after insertion using a stereoscopic microscope (Axio Imager Z2, Zeiss, Germany), frozen section analysis, or anterior segment optical coherence tomography (AS‐OCT, TowardPi Medical Technology Ltd, China).

### Cytocompatibility of the MN Patch

The cytocompatibility of the MN was assessed by evaluating the viabilities of L929 fibrosarcoma cells (American Type Culture Collection, ATCC) in complete medium and leaching medium, as previously reported.^[^ ^41^ ^]^ Briefly, L929 cells were seeded in 24‐/96‐well tissue culture plates (BD Biosciences, China) at a density of 5000 cells/cm^2^ and cultured in either complete medium or a leaching medium containing 2.5 mg mL^−1^ MN patches composed of PVA, PAA‐CD and IR820. Cell activity was initially evaluated through Live&Dead staining (KeyGEN BioTECH, China). Following the manufacturer's instructions, calcein AM (0.5 µL mL^−1^) and ethidium homodimer‐1(2 µL mL^−1^) were diluted in PBS to prepare the staining solution, which was added to the well after the culture medium was removed. The cells were incubated for 30 minutes at 37 °C in the dark. Live cells (green) and dead cells (red) were visualized using an inverted fluorescence microscope (Observer 7, Zeiss, Germany) on Day 1, 3, and 5 of culture. The proliferation of L929 cells was further quantified using CCK‐8 assays (Dojindo, Japan), with absorbance measured at 450 nm using a microplate reader (Synergy H1, Biotek, USA) on Day 1, 3, and 5. Each reported value was the average of at least five measurements.

### Material Histocompatibility of the MN Patch

The biocompatibility of MN was further evaluated using an ex vivo murine model involving MG explants, as previously described.^[^ ^29^ ^]^ Tarsal plates were isolated from untreated eyes of euthanized mice and were then divided into two segments, each containing an equal number of MGs. These segments were randomly cultured in 24‐well culture plates with the conjunctival side up. The culture medium was either defined keratinocyte serum‐free medium (DKSFM; 10744‐019; Thermo Fisher Scientific, Waltham, MA) as a control (CTR) or a leaching medium containing 2.5 mg mL^−1^ MN patches, which consisted of PVA, PAA‐CD, and IR820 for a duration of 24 h. The CCK‐8 assay was subsequently performed following the manufacturer's instructions.

### Photobiological Safety Tests In Vivo

To assess the biosafety of NIR irradiation at 808 nm, normal mice were randomly assigned to either the control group (NIR‐) without irradiation or the treatment group (NIR+) receiving eyelid NIR irradiation every three days for 5 minutes. After 3 months of treatment, corneal and retinal structure and morphology were evaluated. All Mice were anesthetized, and examinations were conducted using a slit‐lamp microscope (SL‐D7, Topcon Inc., Japan) and full field optical coherence tomography (FF‐OCT, Ophthalmic Engineering Research Center, Zhongshan Ophthalmic Center, Sun Yat‐sen University). Corneal images under both white and cobalt blue light were captured. Additionally, the structure of corneal epithelial, stromal, and endothelial cells was analyzed. Pupils dilation was induced with tropicamide phenylephrine eye drops (Santen Pharmaceutical Co., Ltd., China). Fundus images, centered on the optic nerve head, were acquired using optical coherence tomography (OCT) and optical coherence tomography angiography (OCTA) with a specialized small‐animal retinal multimodal imaging system (Robotrak., China). OCT imaging employed a 50°×50° field of view (FOV) with 512 × 512 pixels, repeated 5 times to enhance the signal‐to‐noise ratio (SNR). OCTA imaging utilized the same FOV and pixel configuration as OCT, with 5 repeated scans to optimize SNR.

### HFD Induced MGD Model and Groups

To induce MGD, the mice were fed a high‐fat diet (HFD, 60 kcal% fat, Research Diets, New Brunswick, NJ, USA) for three months, as previously described.^[^ ^35^ ^]^ The control group, designated as “normal”, was fed a standard‐fat diet (SFD, 12 kcal% fat, KERONG, Shanghai, China). Body weight and food intake were monitored throughout the study period. After three months, the HF mice were randomly allocated to four groups (n = 6 per group): untreated control (CTR), blank MN patch treatment (MN), oral administration of ROSI (10 mg kg^−1^) (ROSI), and ROSI‐loaded MN patch (ROSI‐MN) administered every three days. For the MN and ROSI‐MN groups, 5 × 1‐needle‐sized MN patches were inserted into the shaved eyelid skin of anesthetized mice. Mice in these groups also received NIR irradiation (808 nm, 1 W/cm^2^) over closed eyelids for 5 min before being patch removed. All mice were sacrificed 12 weeks post‐treatments for further analysis.

### Gross Clinical Evaluations

Prior to sacrifice, the mice were weighed and photographed at both macroscopical and microscopical levels. In vivo images of corneas, upper eyelid margins, and MGs, as well as ex vivo images of MGs were captured using a slit‐lamp microscope. Corneal epithelial defects were highlighted using 1% fluorescein sodium (Sigma‐Aldrich) staining under cobalt blue light. The areas stained with fluorescein and the total corneal areas were analyzed using imageJ software (Version 2.3.0, the National Institutes of Health), and the percentages of these areas were statistically analyzed. Infrared images of MGs ex vivo were also captured, and the relative MGs coverage in relation to the tarsal plates from different groups were quantified and analyzed.

### Tear Secretion Measurements

Tear production was evaluated using phenol red cotton threads (Jing Ming, Tianjin, China), following a previously established method.^[^ ^35^ ^]^ Topical anesthesia with 0.5% proparacaine hydrochloride (Alcon, USA) was applied to the corneal surface, and any excess fluid was gently removed. After a 5‐min waiting period, a phenol‐red thread with a 1‐mm bend end was positioned in the lower conjunctival fornix, approximately one‐third of the lower eyelid distance from the lateral canthus, for 15 s. Upon contact with tears, the thread changed color from white to yellow‐orange, and then yellow and finally red. The thread was removed after 15 s, and the length of the red‐stained portion was measured in millimeters.

### Serum Cholesterol Measurements

Blood samples were collected via cardiac puncture from the mice in both the Normal and HF group at the time of sacrifice. Samples were allowed to stand at room temperature for 1 h, and then refrigerated at 4 °C overnight. Following centrifugation at 3000 rpm min^−1^ for 10 minutes, the supernatant, referred to as serum, was isolated for cholesterol concentration measurement. This analysis was conducted using a commercially available total cholesterol kit (BC1985, Solarbio, Beijing, China) in accordance with the manufacturer's instructions.

### Histology and Immunofluorescence Assays

After clinical evaluations, the mice were sacrificed, and tissues, including eyelid tissues, eyeballs, and various organs (lymph node, liver, kidney, heart and spleen), were collected. These tissues underwent fixation in 4% paraformaldehyde (PFA), dehydration, and embedding in either optimal cutting temperature (OCT) compound or paraffin. Cross sections, 8‐µm‐thick, were then sliced into prepared and stored at −20 °C (for frozen sections) or at room temperature (for paraffin sections). For staining, Oil Red O solution (Servicebio, Wuhan, China) was applied to frozen sections as described in the literature.^[^ ^42^ ^]^ Paraffin sections were stained with H&E and Masson's trichrome stain (Servicebio, Wuhan, China) following the manufacturer's instructions. Non‐fluorescent sections were examined using a light microscope (Eclipse 50i; Nikon, Tokyo, Japan). Immunofluorescence staining involving fixing paraffin sections in 4% PFA for 10 min, followed by permeabilization and blocked with 5% donkey serum and 0.3% Triton X‐100 in PBS for 1 h at room temperature. The sections were then incubated overnight at 4 °C with primary antibodies diluted 1:100, targeting Keratin14 (Proteintech‐cn, Wuhan, China), PPAR‐γ (Proteintech‐cn), MMP‐9 (Proteintech‐cn), and IL‐1β (Servicebio). After three PBS washes, sections were treated with fluorescein‐conjugated secondary antibodies (1:500, Cell Signaling Technology, MA, USA) for 1 h at room temperature and counterstained with DAPI (Invitrogen, CA, USA). For the evaluation of end‐stage apoptosis, TUNEL assays were performed on paraffin sections using TUNEL FITC Apoptosis Detection Kit (Vazyme Biotechnology, Nanjing, China) according to the manufacturer's instructions.^[^ ^43^ ^]^ Fluorescence images were captured using a fluorescence microscopy (Axio Observer 7, Zeiss, Germany), and the intensity of fluorescence was quantitative analyzed using Image J software.

### Western Blot Analysis

Isolated MGs were homogenized and lysed in cold RIPA buffer containing 1% phosphatase and protease inhibitors (Thermo Fisher Scientific). Protein concentrations were determined using the BCA kit (Pierce Biotechnology, Rockford, USA). For analysis, 30 µg of protein from each group was separated on 12% tris‐glycine SDS polyacrylamide gels and transferred to 0.22 µm PVDF membranes (Millipore, MA, USA). The membranes were blocked in 5% non‐fat milk for 1 h, then incubated overnight at 4 °C with primary antibodies against PPAR‐γ (1:1000, Proteintech‐cn) and GAPDH (1:2000, Proteintech‐cn). After three 10‐min washes, the membrane was exposed to HRP‐conjugated secondary antibodies (1:5000, Cell Signaling Technology) for 1 h at room temperature. Protein bands were visualized using the FluorS MultiImager (Bio‐Rad Laboratories, Inc., CA, USA) with an enhanced chemiluminescence reagent (ECL, Vazyme).

### Statistical Analysis

Data were expressed as mean ± SEM. Statistical comparisons were made using unpaired *t*‐test, one‐way analysis of variance (ANOVA) or two‐way ANOVA through GraphPad Prism 9.0 software (GraphPad Software Inc, San Diego, CA, USA). A P‐value < 0.05 was considered statistically significant.

## Conflict of Interest

The authors declare no conflict of interest.

## Supporting information



Supporting Information

## Data Availability

The data that support the findings of this study are available from the corresponding author upon reasonable request.
