# Supplementary material for: Photothermal‐Responsive Soluble Microneedle Patches for Meibomian Gland Dysfunction Therapy
Source: Adv Sci (Weinh). 2025 Jan 30;12(11):2413962. doi: 10.1002/advs.202413962 (PMC11923895; doi:10.1002/advs.202413962)
Supplement: Supplementary file 1 — Supporting Information [file ADVS-12-2413962-s001.docx]

**Supporting Information**

**Photothermal-Responsive Soluble Microneedle Patches for Meibomian Gland Dysfunction Therapy**

*Fei Yu^2,3#^, Xuan Zhao^2#^, Qian Wang^2,4#^, Yifei Niu^2^, Peng Xiao^2^, Jinze Zhang^2^, Keyi Fei^2^, Yuancong Huang^2^, Liu Liu^2^, Po-Han Fang^2^, Xinyue Du^2^, Weihua Li^2^, Dalian He^2^, Tingting Zhang^2^, Saiqun Li^2^*, and Jin Yuan^1^**

^1^Beijing Tongren Eye Center, Beijing Tongren Hospital, Capital Medical University, Beijing Key Laboratory of Ophthalmology & Visual Sciences, Beijing, 100730, China.

^2^State Key Laboratory of Ophthalmology, Zhongshan Ophthalmic Center, Sun Yat-sen University, Guangzhou, 510623, China.

^3^Sun Yat-sen Memorial Hospital, Sun Yat-Sen University, Guangzhou, 510020, China.

^4^National Clinical Research Center for Ocular Diseases, Eye Hospital, Wenzhou Medical University, Wenzhou, 325027, China.

^#^ Fei Yu, Xuan Zhao and Qian Wang contributed equally

* Corresponding Author:

E-mail: Saiqun Li, lisq9@mail.sysu.edu.cn; Jin Yuan, yuanjincornea@126.com

**
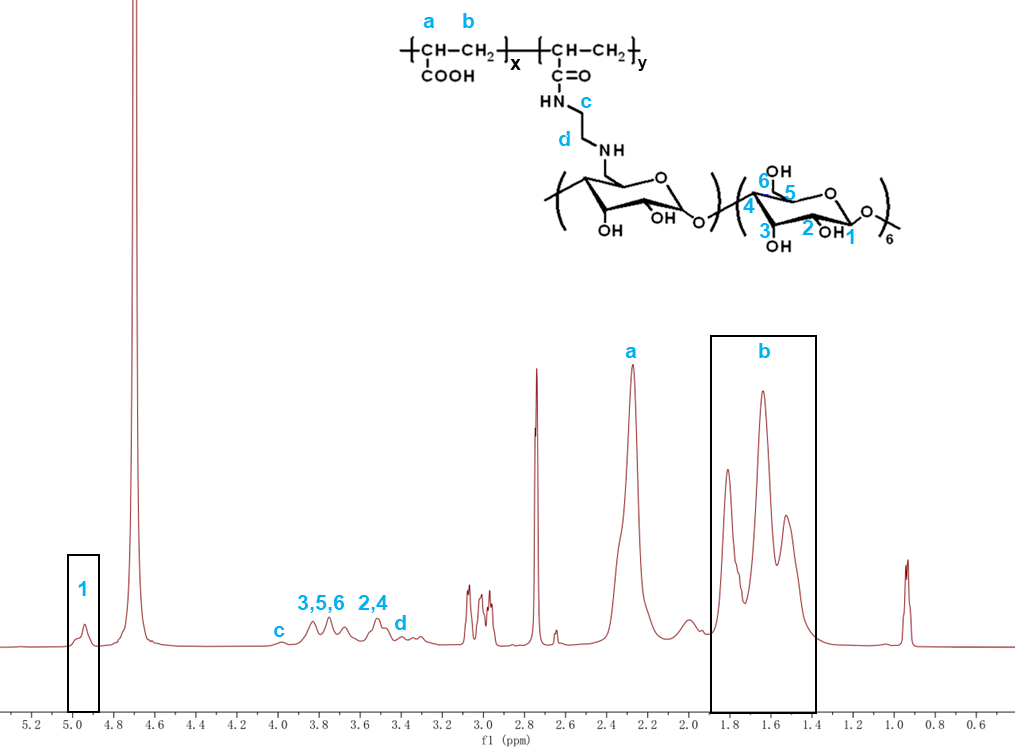
**

**Figure S1.** ^1^H-NMR spectra of PAA-CD.


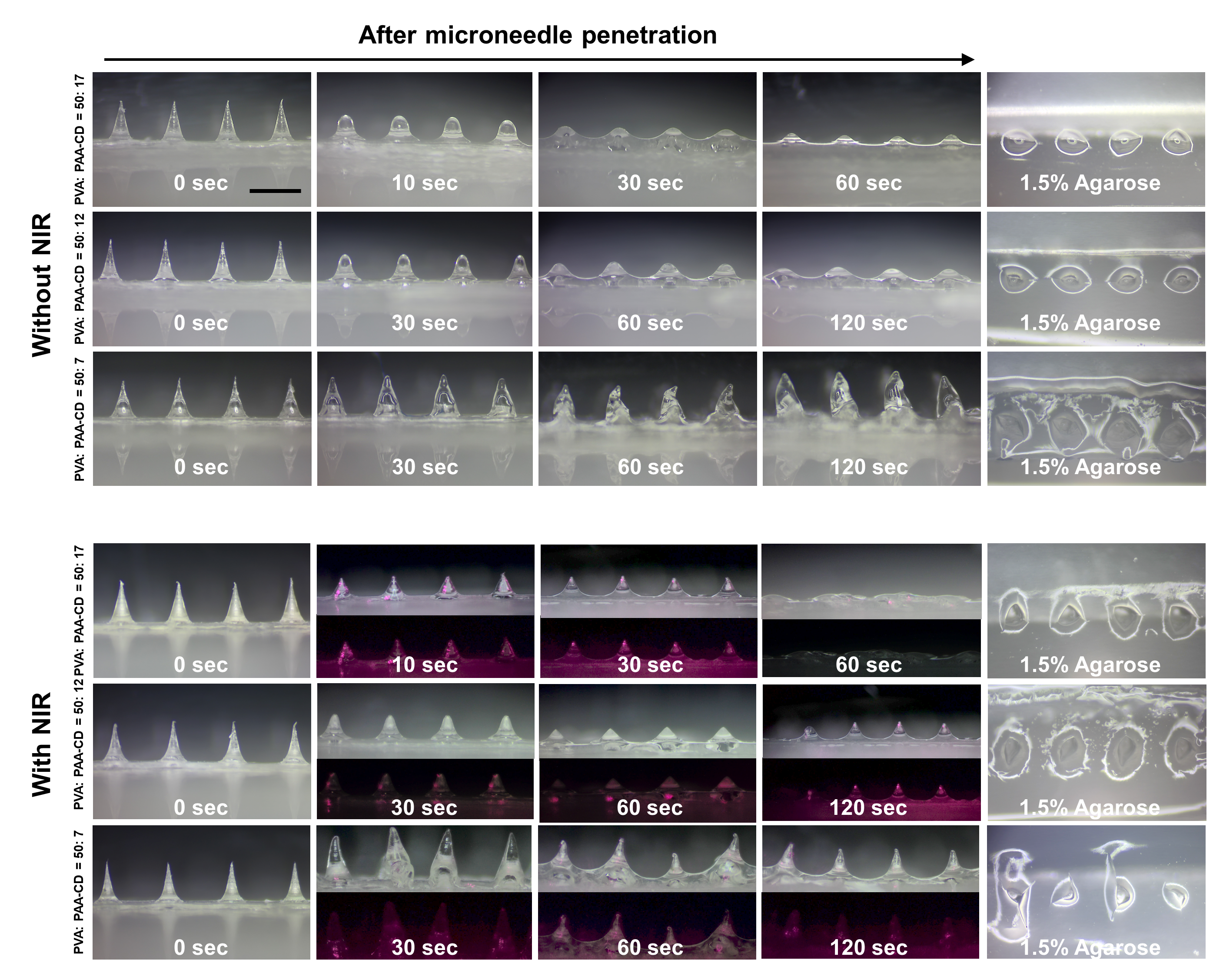


**Figure S2.** Dissolution behavior of MN patches after insertion into 1.5% agarose hydrogel without and with NIR irradiation. Scale bar: 500 μm.


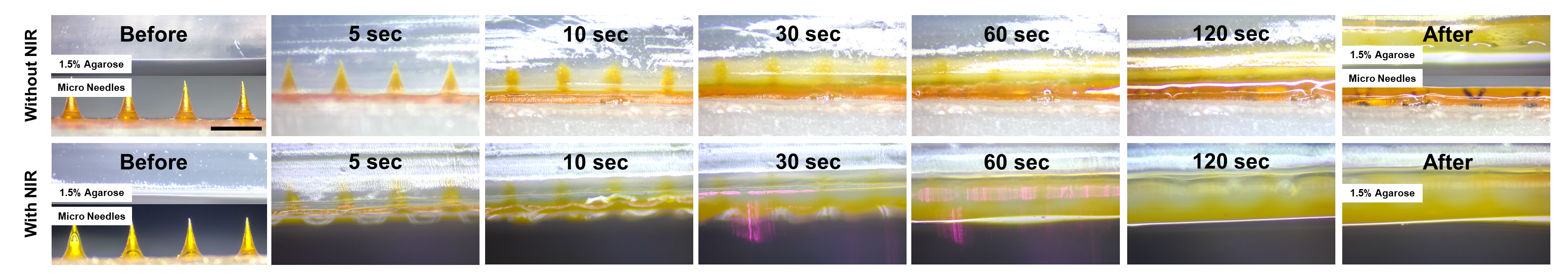


**Figure S3.** Diffusion of MN patches after insertion into 1.5% agarose hydrogel without and with NIR irradiation. Scale bar: 500 μm.


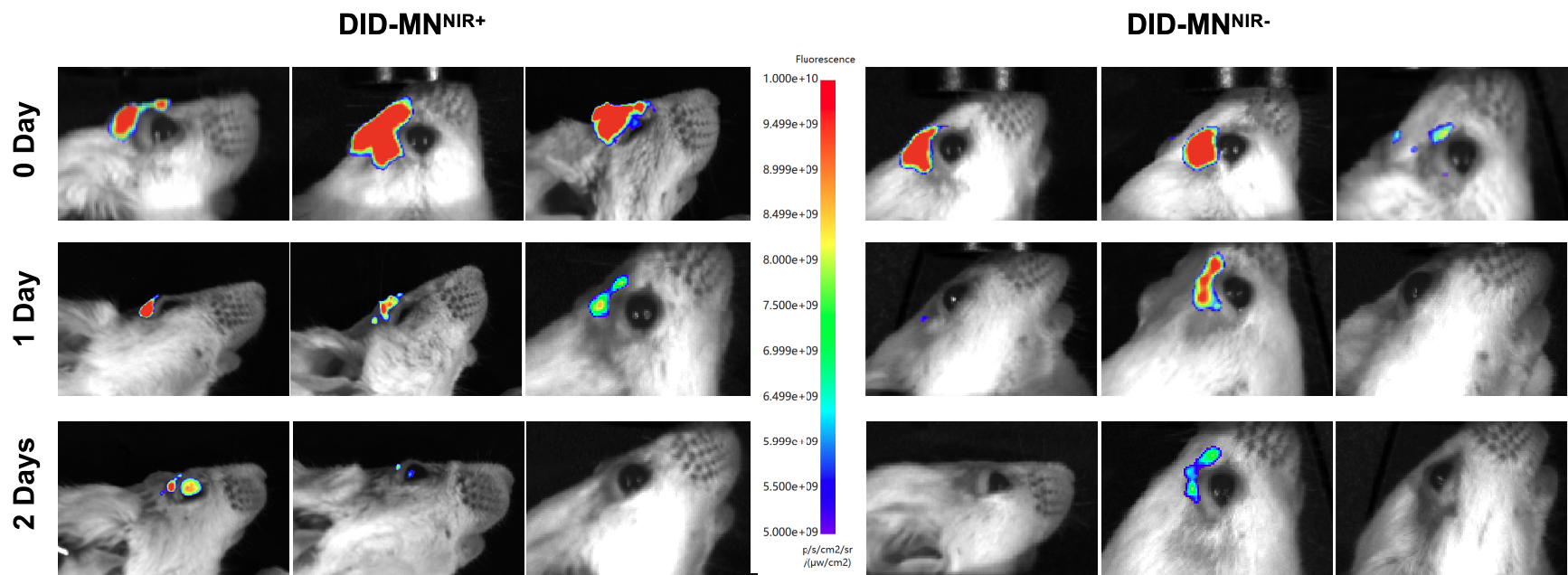


**Figure S4.** Representative monocular side IVIS images at specified time points after treatments with the CM-DID loaded MN patch either with NIR irradiation or not.


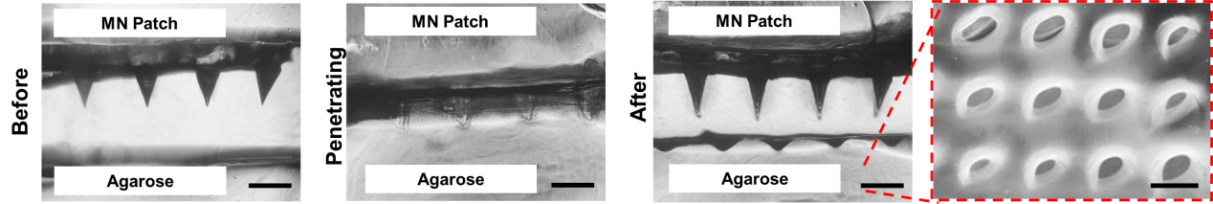


Figure S5. Representative bright-field microscopy images of the MN patch before, during and after insertion into agarose hydrogel. The red dashed box showed the enlarged image of microchannels left in agarose hydrogel after insertion. Scale bar: 500 μm.


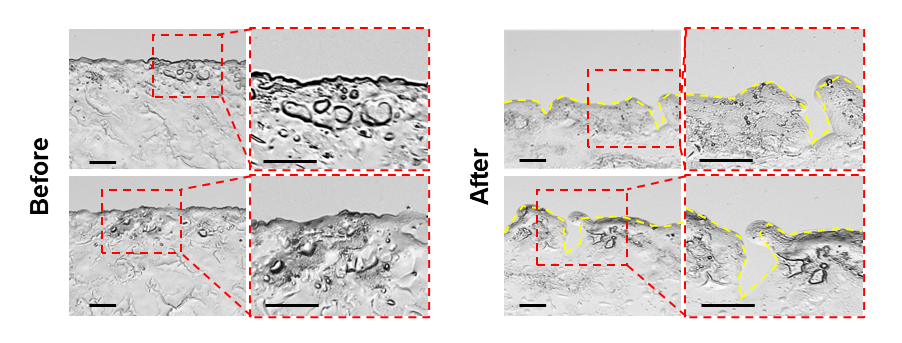


**Figure S6.** Representative frozen section images of murine back skin before and after insertion. The red dashed box displayed specific enlarged areas. The yellow dashed lines highlighted the surface contour of the skin. Scale bar: 200 μm.


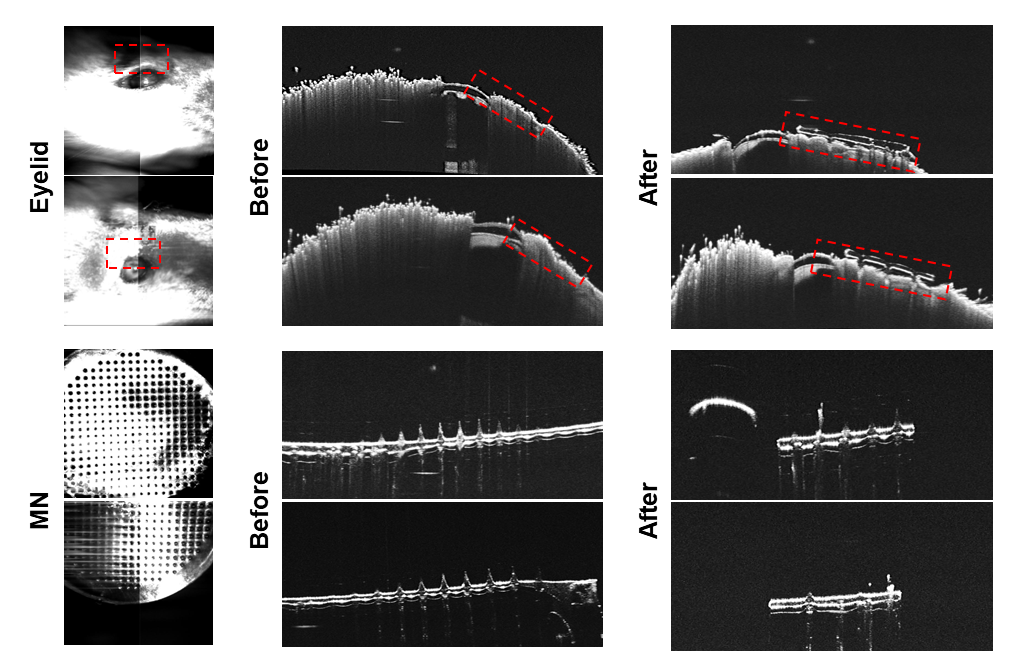


**Figure S7.** Representative AS-OCT images of the murine eyelid and the MN patch before, during and after the insertion. The red dashed box showed the location of the shaved murine upper eyelid.


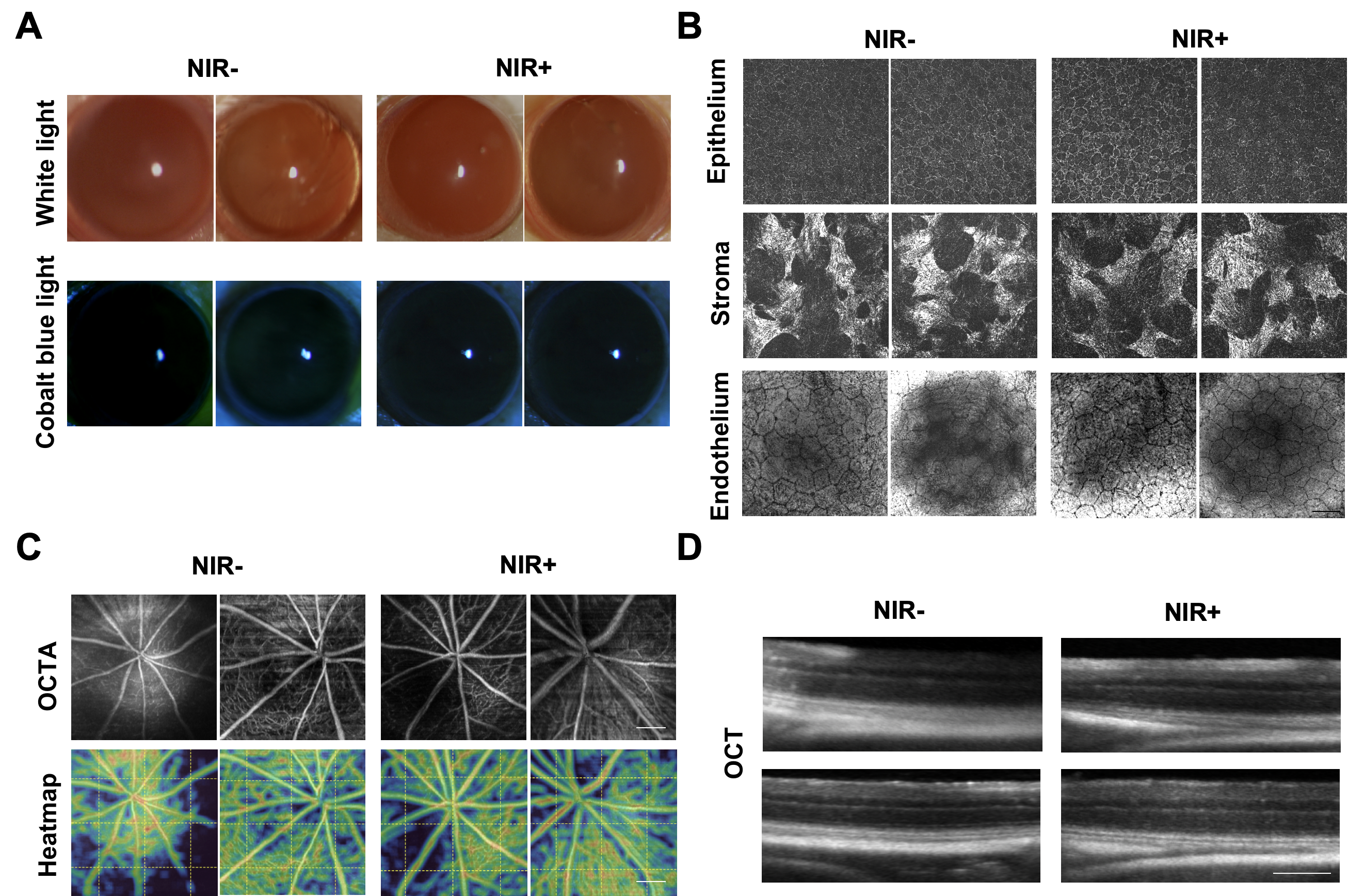


**Figure S8.** (A) Representative bright field (upper) and fluorescein staining (lower) images depicting corneal opacity and integrity of corneal epithelium in NIR- and NIR+ groups. (B) Representative images of FF-OCT to measure the corneal cellular morphology and density at different layers. Scale bar: 25 μm. (C) Representative OCTA (upper) and heatmap (lower) images of fundus manifesting central retina vessels from two groups. Scale bar: 100 μm. (D) Representative OCT images of transverse sections of retinas from two groups. Scale bar: 100 μm.


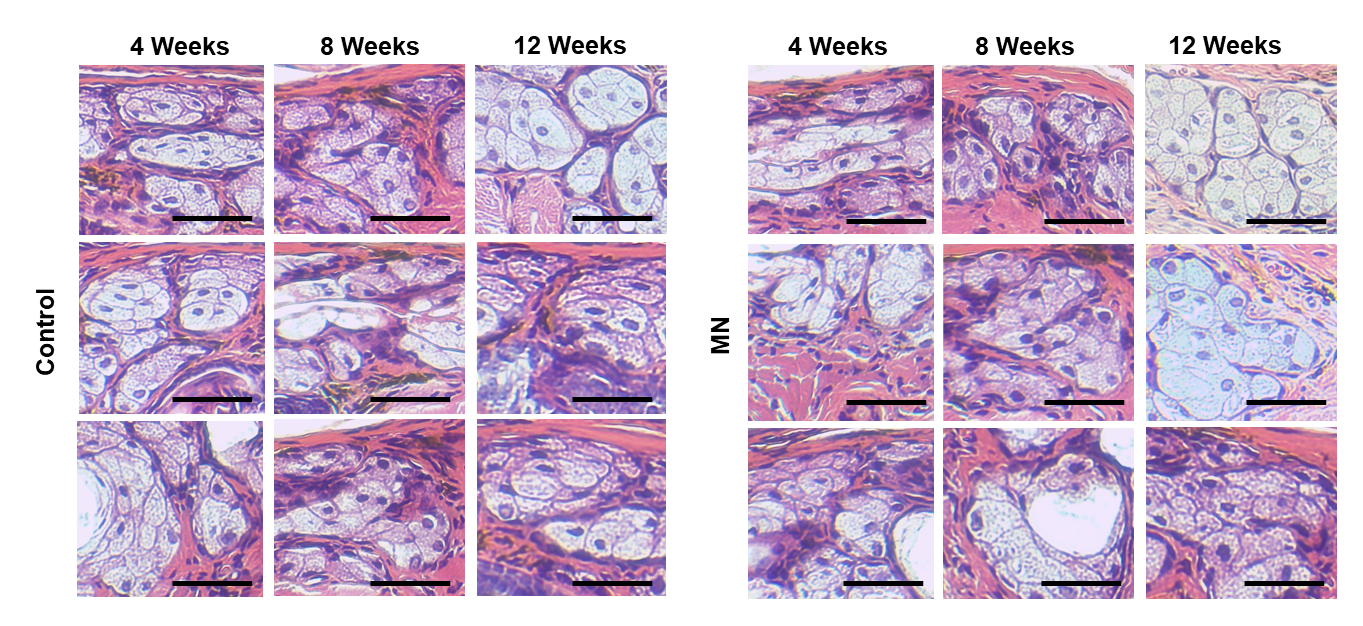


**Figure S9. The biosafety of the MN patch.** Representative H&E images of MGs from the no treatment (Control) and blank MN treatment (MN) groups at specified timepoints. The blank MN patch with 5×1 needles was applied to the upper eyelid skin of mice every three days for three months. Scale bar: 100 μm


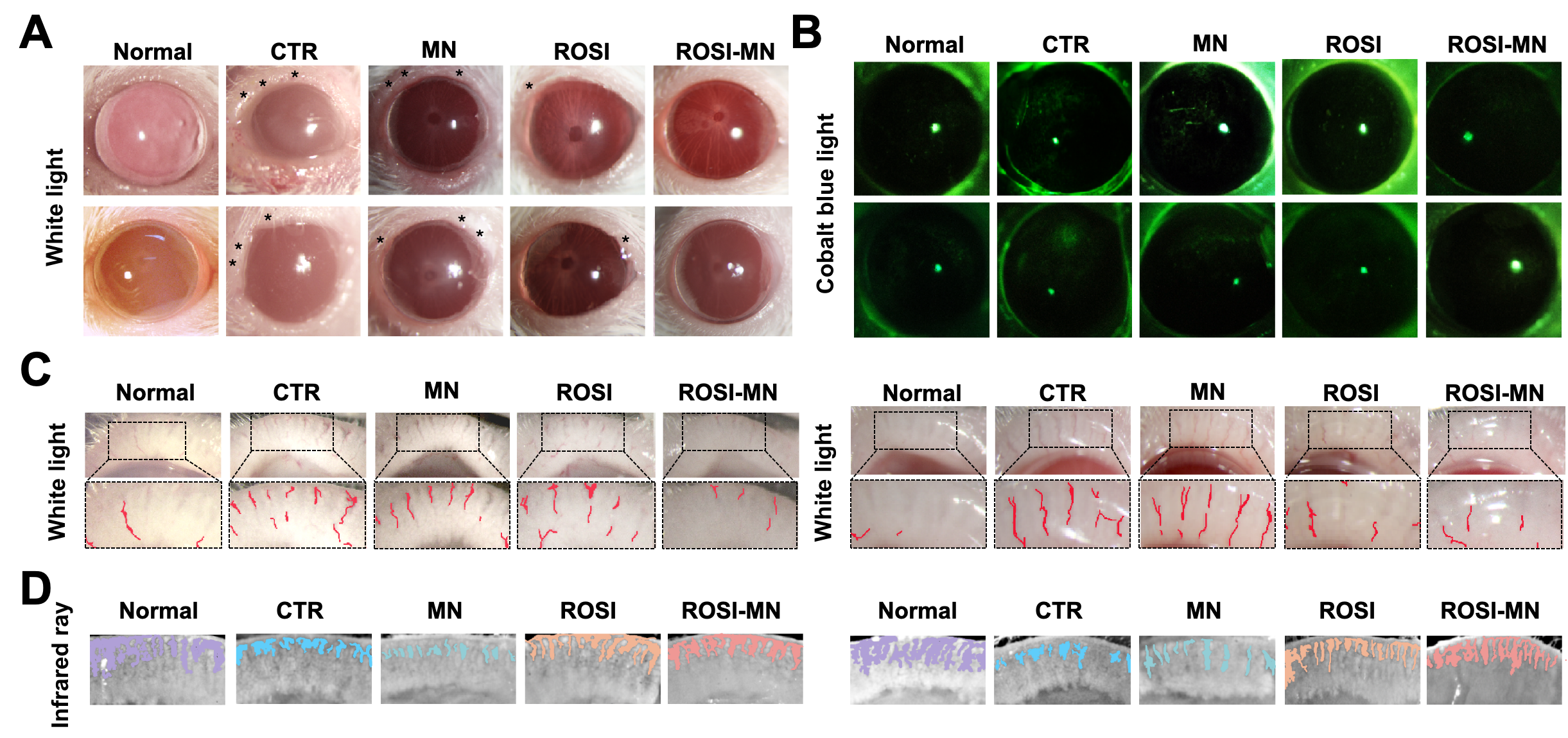


**Figure S10.** (A) Representative gross images of ocular surface showed the morphology of eyelid margins and corneas in mice. Black stars indicated areas with obvious anomalies. (B) Representative fluorescein staining images of corneas demonstrating the epithelial defects in each group. (C) Representative *in vivo* bright field images of MGs. The black dashed line represented enlarged views of central areas and the red areas highlighted capillaries. (D) Representative infrared images of MGs *ex vivo*. Colored areas represented the gaps between meibomian glands.


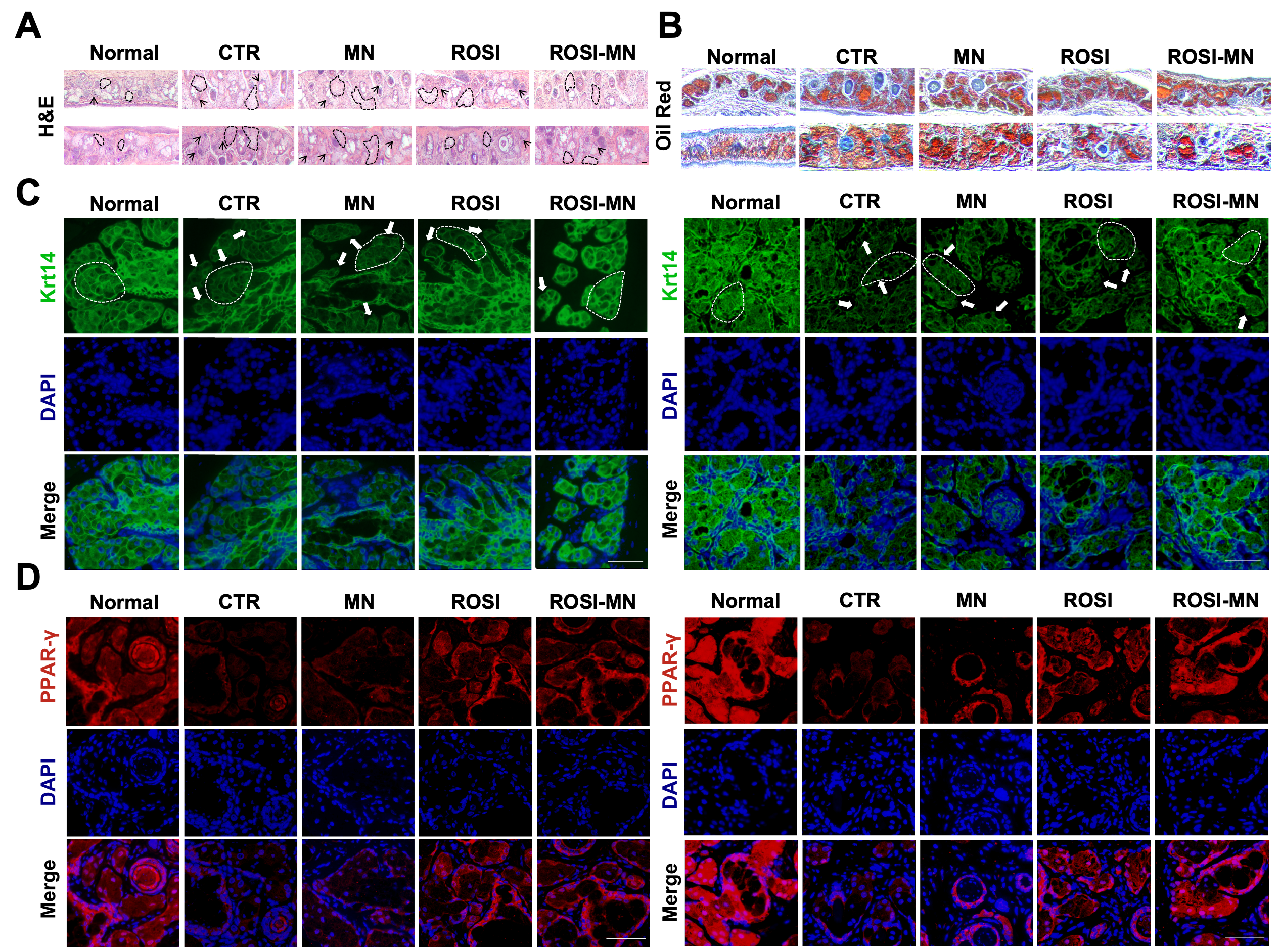


**Figure S11.** (A) Representative H&E staining showed the microstructure of MG acinus from each group after 3-month treatments. The black arrows pointed towards monocytes and the dotted line protruded the shapes of acinus. Scale bar: 50 µm. (B) Representative Oil Red O staining images of MGs indicating lipid areas of MGs in each group. Scale bar: 50 µm. (C) Representative Krt14 (green) staining images of MGs. White arrows showed the destroyed acini membrane and the dotted white line protruded the shapes of acinus. Scale bar: 50 µm. DAPI stained nuclei (blue). (D) Representative immunofluorescent staining images of PPAR-γ (red) in the MGs. Scale bars: 50 µm. DAPI stained nuclei (blue).


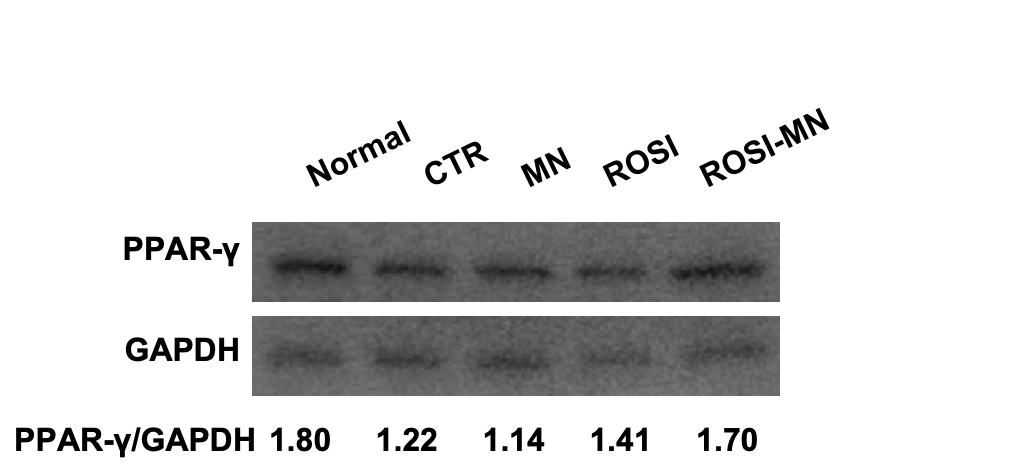


**Figure S12. The treatment of ROSI-MN increased the expression of PPAR-γ in MGs**. WB analysis was performed to compare the expression of PPAR-γ in MGs after different treatments. The grayscale ratios of protein expression was listed below. The result confirmed that the expression of PPAR-γ decreased after long-term HFD feeding, and both ROSI and ROSI-MN treatments could enhance the expression of PPAR-γ.


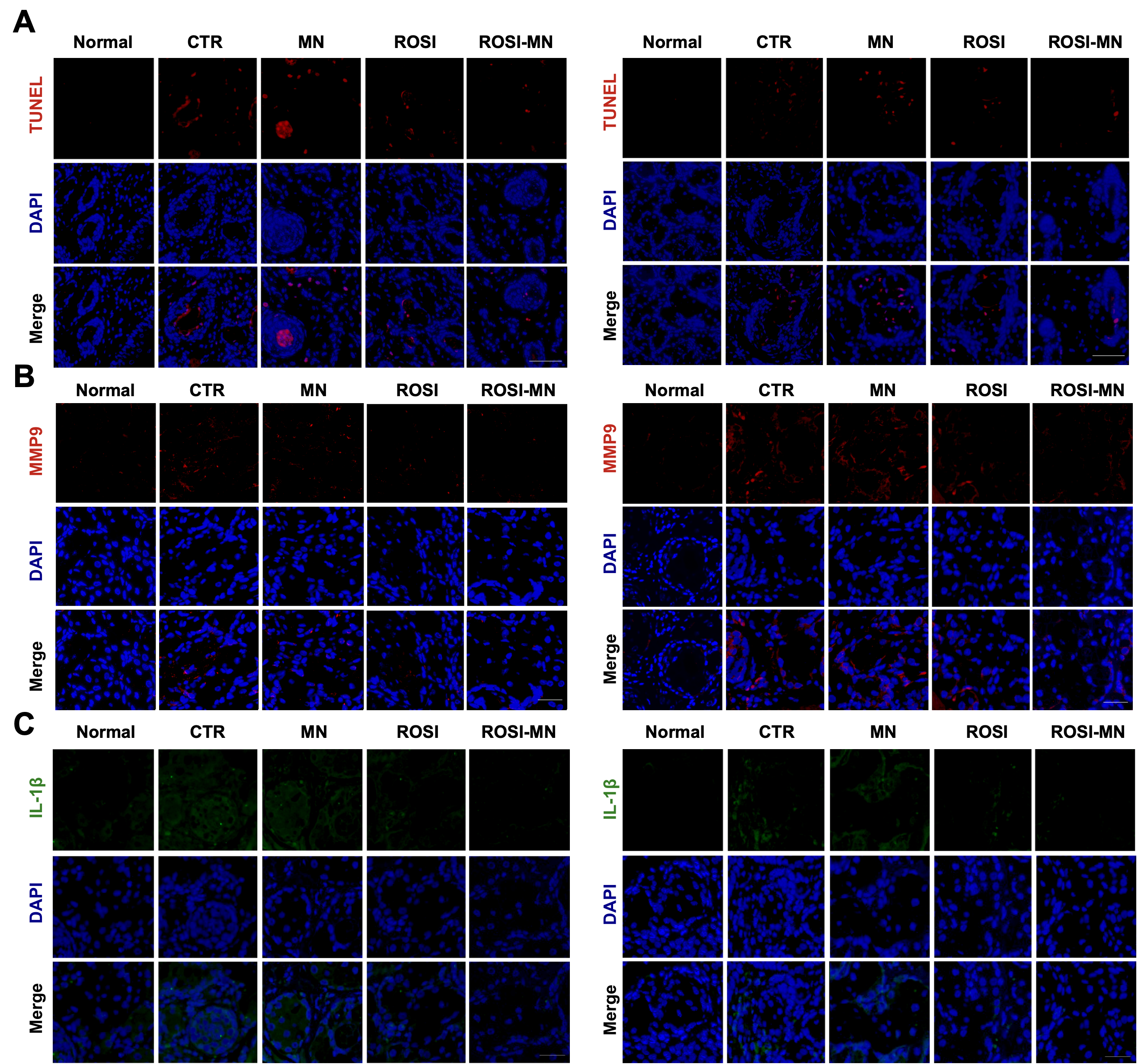


**Figure S13.** (A) Representative images of TUNEL (red) staining in MGs. The cell nuclei were stained blue with DAPI. Scale bar: 50 µm. (B) Representative images depicting MMP9 (red) immunofluorescent staining in MGs. DAPI (blue) was used for nuclear staining. Scale bar: 50 µm. (C) Representative IL-1β (green) immunofluorescent staining images of MGs. DAPI (blue) was used for nuclear staining. Scale bar: 50 µm.


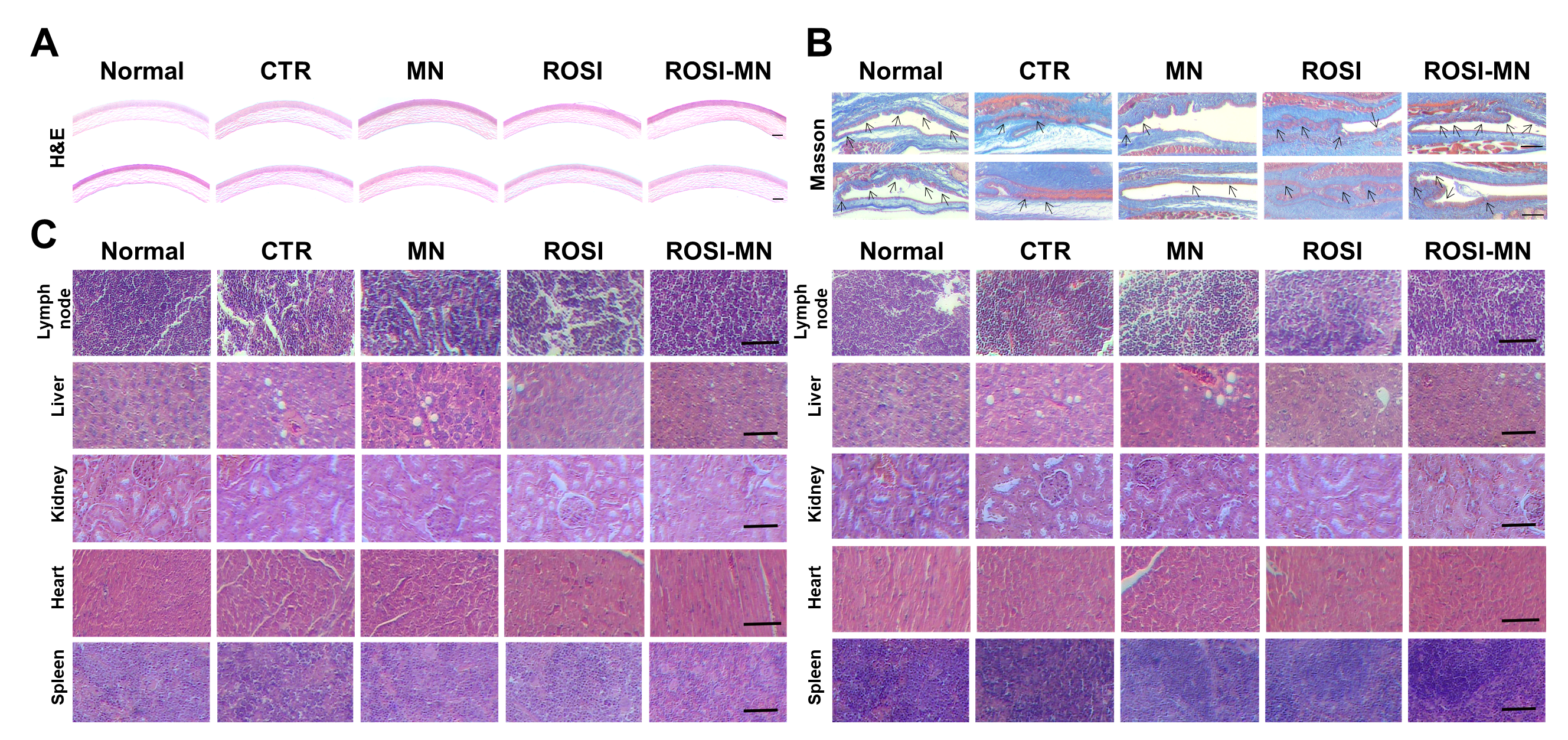


**Figure S14.** (A) Representative images of H&E staining of corneas. Scale bar: 50 μm. (B) Representative Masson's trichrome images of the conjunctiva. Black arrows indicated the goblet cells. Scale bar: 50 μm. (C) Representative images of H&E staining of cervical lymph node, liver, kidney, heart and spleen from five groups. Scale bar: 50 μm.
